# HF Radar Signatures and Their Use for Target Classification, Recognition and Identification

**DOI:** 10.3390/s26051412

**Published:** 2026-02-24

**Authors:** Stuart Anderson

**Affiliations:** Physics Department, University of Adelaide, Adelaide 5005, Australia; stuart.anderson@adelaide.edu.au

**Keywords:** HF radar, over-the-horizon radar, target classification, radar signatures

## Abstract

**Highlights:**

HF radar provides efficient, wide-area surveillance of air and surface vehicles at over-the-horizon ranges, but it is often the case that knowing the mere presence of a ship or aircraft has limited value. In order to assess any situation and take appropriate action, the radar operator needs information on the types of vehicles involved, whether they belong to known classes, and, ideally, their unique identities. The extent to which a radar can deliver such information depends not only on radar design but also on the depth of understanding of the physics responsible for the vehicle radar signatures, together with radar operating procedures and signal processing architectures that retrieve the target information.

**Main findings**

- a semantic framework for discussing target characterization
- a taxonomy of target signature domains and characterization methods
- an examination of the physics underlying each method, with illustrative examples

**Implications**

The results of this paper will support HF radar operational effectiveness in many domains, including:

- the recognized air picture (RAP) and recognized surface picture (RSP)
- early warning and threat assessment
- over-the-horizon targeting
- radar resource allocation

**Abstract:**

The challenging goal of equipping HF radars with a target classification ability has been pursued for many years, yet no satisfactory system-level methodology has been reported. This shortcoming severely limits the utility of radar information as, without knowing the nature of detected objects, there is little prospect of understanding the situation and tailoring a suitable response. In this paper, we present a framework within which a comprehensive approach to target characterization can be formulated. We proceed to explore a wide range of physical mechanisms whereby target information is impressed on HF radar echoes, illustrated with real data. This paper concludes with a commentary on the difficulty of integrating target classification, recognition, and identification procedures with other radar tasks and resource management.

## 1. Introduction

Recent years have seen a major resurgence of interest in HF skywave ‘over-the-horizon’ (OTH) radars [1,2,3,4,5,6,7,8,9,10,11,12], along with modest growth in the number and geographical distribution of radars exploiting HF surface wave and line-of-sight propagation mechanisms, sometimes in hybrid configurations [13,14]. This proliferation may be due in part to the need for greater autonomy in the event of frayed geopolitical alliances, but it is primarily a response to the increased speed and range of weapons and platforms. In this context, the four key advantages of HF radar technology—wide area OTH coverage, persistent surveillance, the ability to function autonomously, and a unique capacity to probe deeply into physical processes on and under the ocean surface—offer a practical and affordable solution.

Defense users of OTHR-derived information remain primarily concerned with maintaining a recognized air picture (RAP) or recognized maritime picture (RMP), while others focus on addressing a diverse spectrum of civil applications related to merchant ship traffic distributions, Exclusive Economic Zone management, and remote sensing of surface currents and sea state, especially to support scientific and ocean industry activities [15]. Beyond these established applications, there is growing awareness of the prospective exploitation of increased radar sensitivity and accuracy, coupled with robust, high-data-rate communications, to enable real-time combat support missions such as engagement set-up, advice to platforms for optimizing their onboard sensors, close vectoring of intercepts, control of jammer assets, and battle damage assessment.

But there is a catch. Even with a comprehensive picture of the spatial distribution of platforms, few of these tactical missions can be accomplished without the ability to classify the various players—assets, neutrals, or adversaries—in the surveillance zone. Rules of engagement, choice of targets, selection of weapon systems, and activation of supporting electronic measures all rely on acquiring adequate knowledge of the type and affiliation of the participants. This task—most commonly known as target recognition or classification, though we shall refine this terminology—has not attained anywhere near the same level of maturity as the precursor task of detection or the ongoing tasks associated with tracking.

In order to exploit all avenues that yield information that can contribute to the characterization of echo sources, HF radars need to look beyond the intrinsic, free-space electromagnetic scattering properties of candidate targets. They must take into account the constraints imposed by the HF channel, including the prospect of modulation impressed on the radiated signal during propagation to and from the target, as well as the interaction of the target with its immediate environment. In the latter case, the interactions may involve a combination of mechanical, hydrodynamic, and electromagnetic coupling mechanisms. As a consequence, a deep understanding of the physics of the entire observation process lies at the heart of successful target characterization at HF. Nowhere is this more critical than in the case of radar configurations that involve skywave propagation, but, as will be explained, HF surface wave propagation too introduces challenging complications that are absent from free-space propagation.

This understanding of the radar process physics must be supplemented by an accurate representation of the properties of the radar itself. Bounds on access to the HF spectrum, either from hardware limitations or from imposed regulatory constraints, are the primary determinants of the feasibility of many classification techniques. Most commercial HFSWR systems are deployed with a single frequency band just wide enough to accommodate the radar waveform, whereas some military-grade radars are free to operate over a substantial fraction of the HF spectrum. Practical issues such as timing accuracy, spectral purity, presence of system nonlinearity, spurious-free dynamic range, precise knowledge of the array manifolds, and calibration of the signal path lie at the heart of successful extraction of the more subtle features of the target information embedded in the echoes. In addition, radar siting can play a decisive role in mission performance, as we explore later in this paper. Ideally, the intention to equip an HF radar with a target classification capability, not merely a detection capability, should be taken into account at the design stage, not treated as an option that can be added later.

In this paper, we set out to demonstrate that the multiplicity of target signatures accessible to a suitably designed HF radar, augmented in some cases with contextual information, have the potential to support a practical, robust target classification capability in operational radar systems. We begin, in the following section, by reviewing previous research, noting the limitations of some proposed schemes that ignore crucial aspects of operational implementation. Then, in Section 3, we present a lexicon that formalizes and clarifies the hierarchy of classification objectives, along with a practical definition of radar signatures that supports an integrated approach. This is important because many papers in the open literature misleadingly treat the terms classification, recognition, and identification as synonyms. Further, in order to represent the entire observation process, it is necessary to provide the connection between what happens in the scattering zone with the radar observables that are the input to the classification procedure; this is the function of the radar process model framework which we review briefly.

Next, in Section 4, we survey the diversity of physical mechanisms by which target information is encoded in the scattered radar signals in ways that can, potentially, lead to characterization of the target by appropriate signal processing; we organize these in a taxonomy that accommodates all these techniques and could be adapted to new methods if or when they are developed. Some of these mechanisms are obvious, at least superficially, but others are subtle and their successful exploitation can be highly dependent on the details of radar design. Wherever possible, we present examples of these phenomena, obtained with operational or experimental HF radars, both skywave and surface wave; where data is not available (or releasable), we substitute results generated by state-of-the-art computational models. As our goal is to advance the field, not only by presenting some new and promising techniques but also by providing a foundation that others might find helpful, we have tried to explain the phenomenology in considerable detail. Section 5 outlines some of the operational and environmental constraints, along with the need for enhanced auxiliary support systems. We foresee a role for artificial intelligence, though how this might be implemented is a blank canvas. A key point is that selection of effective signatures is intimately coupled with prevailing propagation conditions and competition for resources. Our conclusion, supported by many years of experience with HF radars, is that target classification with present-day HF radar technology is achievable much of the time with accuracy and availability compatible with operational requirements.

## 2. Previous Research

Early research on HF radar cross section (RCS) and its relation to target type can be traced to World War II, when the British Chain Home radars were being designed to detect German aircraft over Europe and the approaches to Britain. The choice of transmitter frequency and antenna polarization were driven primarily by the expectation that alignment of the electric field with the wings of aircraft at the half-wave dipole resonance would maximize the return and enable discrimination between bombers and fighters. Later, during the Cold War, the HF RCS of aircraft, ballistic missiles, and other vehicles was studied, along with techniques for reducing RCS by impedance loading. Experiments with HF skywave radars operated by the Stanford Research Institute and the Naval Research Laboratory in the US during the 1960s yielded crude estimates of the RCS of several ships and aircraft [16,17], while the use of sea clutter as a prospective reference calibrator was proposed in the same era [18]. Many other HF radars were deployed, but the goal of demonstrating a meaningful target classification capability was never achieved with those systems. Studies with an HF surface wave radar on San Clemente Island in the 1970s reported measurements of the skin echo RCS of small vessels at frequencies spanning much of the HF band, and these confirmed the relevance of the principal vertical dimension of the target to the appearance of resonances in the RCS, with implications for target classification.

As the US expanded studies that led to the development of the USAF OTH-B skywave radars, scientists at the Ohio State University ElectroSciences Laboratory conducted extensive anechoic chamber measurements of scale models, using gigahertz frequencies, to map the variation of HF RCS with aspect and frequency [19,20]. In later studies, the full polarization scattering matrix was recorded; this data was combined with simple Gaussian noise models to evaluate the performance of classifiers based on access to calibrated multi-frequency and multi-aspect inputs. Both supervised and unsupervised classifiers were tested [21,22,23,24,25,26,27,28,29,30]. The general conclusion drawn from these studies affirmed the feasibility of a meaningful classification capability for a modest number of target types, typically 6–10, provided that the measurements were calibrated in absolute units. What these studies did not do was examine, or even identify, the key practical question: how does one calibrate, or at least cross-calibrate, HF radar measurements obtained at multiple frequencies spanning an octave or more, when the propagation channel is itself frequency dependent for both skywave and surface wave propagation. Even more crucially in the skywave case, how does one deal with the added complications of polarization transformation—both repolarization and depolarization—during ionospheric propagation, and is it possible to design wide-band polarimetric antennas at HF with the desired radiation and response attributes? It was not until 1992 that the skywave propagation problem was addressed [31], adopting simple Rayleigh–Rician fading channel models and applying a random scaling factor to aircraft target RCS measurements from the OSU anechoic chamber archive.

Despite the chasm between anechoic chamber measurements and simple channel models and the real challenges of operational implementation, these studies collectively constituted the first detailed analysis of the HF classification task.

All these studies concentrated on the measurement or estimation of attributes of the target skin echo, in some cases guided by calibration via sea clutter; later classification work proceeded to the next step, assessing classifier performance based on the predicted aspect, frequency, and polarization dependence of the echo, though only the magnitudes of the scattering matrix elements were considered.

It scarcely needs to be remarked that computational electromagnetic modeling of air and ship target RCS have been carried out by many other HF radar groups but primarily for calculating detection probability, not addressing the classification and recognition objectives. (One exception is theoretical modeling carried out by NIIDAR in the Former Soviet Union (FSU) in the 1980s, using their in-house methods. A comparison of some of their model results with those carried out in Australia for the same set of American and Soviet fighter aircraft targets, but using the NEC 4 code, revealed generally good agreement, within 1 or 2 dB, with a few interesting systematic departures).

Attention is now turned to the phenomena that collectively determine the propagation channel characteristics so that absolute RCS could be derived. In skywave radar applications, the observable impact of polarization transformation in the ionosphere had earlier been established at SRI [32,33]; more detailed experiments using diversely polarized transponders were carried out in Australia and used to investigate the polarization bandwidth of skywave channels and hence the waveforms that should be used for polarimetric measurements of targets [34]. A more recent experiment, using polarimetric antennas for transmit and receive over a one-way oblique path, [35] explored the separate mechanisms of repolarization and depolarization, revealing the somewhat disconcerting importance of the latter. Inversion of wide-sweep ionograms and networks of oblique and vertical incidence sounders helped improve the fidelity of real-time ionospheric models through which fast ray tracing could be executed, in conjunction with calculation of nondeviative absorption along the ray path. The scattering coefficients of land surfaces were measured, focusing on the identification of local features that could be used for calibration. However, one experiment measured the temporal variations of land scattering coefficients due to seasonal rainfall, finding them to be of the order of 2–3 dB [36], enough to influence target classification schemes based on RCS magnitude alone.

The studies listed above addressed time-invariant scatterers. The generalization to time-varying discrete targets at HF was developed at NRL, following observations of harmonically related modulation sidebands in the Doppler spectra of echoes from helicopters, recorded with the NRL MADRE radar in 1968 [37]. Normally, these maintain a simple relationship with the shaft rotation rate and the number of blades, as expected from elementary physical considerations [38] and treated in more detail in [39]. It was discovered during an experiment in Australia in 1983 that the scattering spectrum can be more complicated, and an important explanation was found, with important implications for target identification, as we discuss in detail in Section 4.

During the 1980s and after, the complex nature of the skywave propagation channel was explored and effective signal processing techniques developed to mitigate many of the deleterious effects [40], but not all, so the accessibility of target classification techniques remains emphatically dependent on the ionospheric weather. In the case of surface wave propagation, polarization transformation is not an issue, but path loss is of vital concern. Calibrated measurements in several countries established the adequacy of standard theoretical models of propagation loss for low sea states, but rough seas were found to be difficult to predict to the required accuracy.

**Figure 1:**
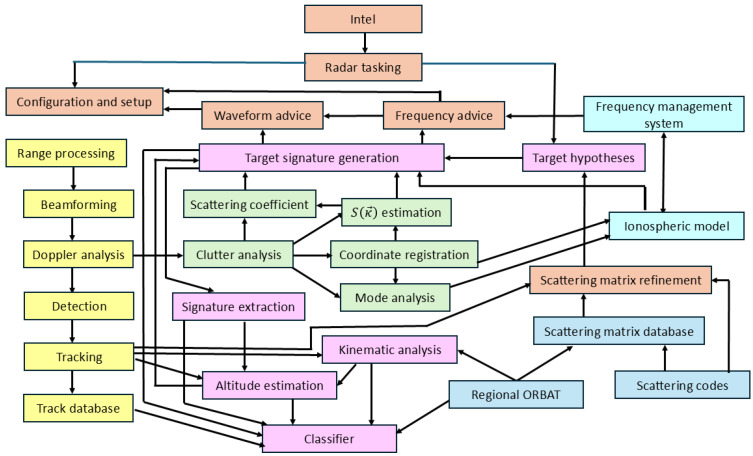
An early design for a target classification scheme, highlighting the transfer of information between radar subsystems, databases, and processing steps [39].

In the late 1990s, the experience gained from decades of experiments with the Jindalee radars was used to design a target classification scheme, though it was never implemented in its entirety; the top-level flowchart of that scheme is shown in Figure 1. An important outcome of that study was confirmation that an architecture supporting connectivity and feedback between radar subsystems and resource management procedures is essential.

An illustrated catalogue of various types of target echo contributions was compiled in a study for a NATO meeting in 2004, along with a detailed lexicography for describing the different levels of classification and guidelines for implementation of operational procedures for capturing the required target echo features [41]. (That report was supposed to remain restricted, but an abbreviated version was leaked to the internet some years later. We include some of that material to make this paper self-contained.) Since then, several additional approaches have been explored; the major new developments have been the rigorous exploration of nonlinear scattering, platform dynamics, and ship wakes as avenues for classification. We shall expand on these in later sections.

Despite the strong motivation to implement a reliable target classification capability in operational radars, with few exceptions the technical challenges have not succumbed to the efforts of the radar researchers, though considerable progress has been made in a number of areas. On the basis of many experiments, much of the relevant physics is now understood, a variety of approaches to differentiating between targets of interest have been conceived and explored by experiment or modeling, concepts for integrating these schemes within the radar tasking and control architecture have been proposed, and many mathematical and computational tools have been devised to model and interpret radar observations. Moreover, the realization that the ability to classify targets depends on the degree of control over radar resources, and radar design, is now taken into consideration when proposing enhancements to existing radar systems.

## 3. Target Characterization: Signatures and the Radar Process Model

### 3.1. The Lexicon of Target Characterization

The first step towards developing an effective target characterization scheme for practical operational use is to establish a lexicon that defines the levels of detail that might be sought. This is not an etymological vanity—it plays a central role in guiding the way the radar should be operated to achieve the desired information retrieval. Important aspects of that guidance include the provision and exploitation of auxiliary information, either derived from the radar itself or imported from external sources. Accordingly, the following terminology, building on usage in the domain of statistical pattern recognition, is adopted in this paper and recommended for operational use. For clarity, the levels are expressed below by their verbs rather than as nouns. Proceeding from the most general level, we have:

***Classify*** *—associate with, or assign to, one of a number of sets (classes) which are distinguished by one or more criteria, irrespective of whether there is any prior knowledge of the class membership or class boundaries*

***Recognize*** *—establish membership of one of a number of disjoint **known** sets (classes). Usually these are labeled by supervized learning from known exemplars*

***Identify*** *—establish the absolute sameness with one of a number of possible individual members of a class of known elements*

Here, we introduce an additional step—*diagnosis*—suggested by recent studies of the parametric dependence of naval vessel echo characteristics:

***Diagnose*** *—extract information about the internal state of a recognized entity*

Further, we have, in the past [41], suggested the possibility of *intentification,* which we define as follows:

***Intentify***—*on the basis of all the retrieved target information, together with the prevailing RAP and RSP, infer the likely mission of the target*

Obviously, some of these steps are highly ambitious objectives, perhaps only rarely achievable. Their attempted execution has direct implications for the allocation of available radar resources and the selection of waveform set, but every step along the chain increases the value of the information, so it would be foolish not to provide a radar management structure capable of addressing all the possibilities.

We need also to bear in mind the precursor stages to target characterization—*detection* and *discrimination*, which isolate those components of the radar echo contributed by the target. Detection scarcely needs definition here, being such a fundamental concept in radar, but for consistency with our approach and completeness, we propose:

***Detect***—*register the presence of an object or disturbance of interest from the response it elicits in the radar*

Discrimination as we apply the term in our approach to target characterization is a little more subtle:

***Discriminate***—*isolate those components of the received signal that arise from, or are modified by, the presence of the particular entity or phenomenon under consideration*

From this definition, it is clear that *discrimination* is a potentially important step. Ideally, the assignment to class should take account of all the target-related energy in the received signal, not just the concentration around the ‘center of mass’ or peak that may have sparked an initial detection as an anomaly discovered by a signal processing operation such as constant false alarm rate thresholding. Yet, in practice, we cannot expect to implement special waveforms and optimum filters to accommodate every possibility, especially when some distributed signatures depend explicitly on the prevailing environmental conditions. Compromises need to be made, and at the present stage of development, almost all exploratory classification schemes begin with threshold detection of localiszd anomalies in some signal decomposition space. The outputs from this simple procedure can then be used to inform a second, more sophisticated process for collating target-related energy.

### 3.2. Target Signatures and the Radar Process Model

Our concern is with those observables that inform on the physical and dynamical attributes of the target. Those observables will depend not only on the intrinsic properties of the target but also on its coupling to the environment and perhaps the presence of neighboring bodies. Thus, at least four types of scattering mechanism can contribute to the signal received by the radar:

(i) scattering from any targets present, including effects resulting from coupling to the environment
(ii) scattering from the neighboring environment, including effects resulting from coupling to targets
(iii) multiple scattering involving both (i) and (ii)
(iv) scattering involving any extraneous signals present on the targets that can convey target information to the radar despite their independent origin

For later reference, we shall name these mechanisms $S_{t}$, $S_{e}$, $S_{m}$, and $S_{x}$.

**Figure 2:**
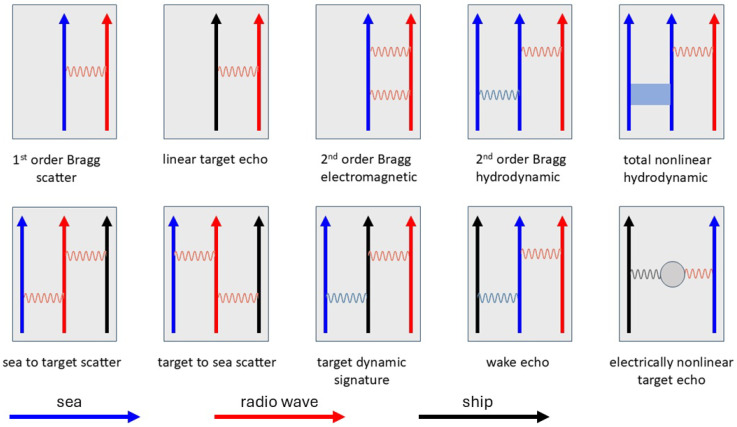
A pictorial representation of scattering mechanisms involved in target classification, loosely based on Feynman diagrams. The vertical axis represents time.

Mechanisms (i)–(iii) seem obvious, but (iv) is less so. Aside from being an independent additive noise source, extraneous signals can, in principle, couple with the incident radar signal due to the presence of electrical nonlinearity in shipboard structures. However, even (i) and (ii) are nontrivial. Ships in rough seas have their motions and attitude modulated by the wave forces, while the ocean surface environment is perturbed by the action of the ships moving through it, creating wakes. These complexities turn out to be fruitful avenues for target characterization. A diagrammatic representation of these and associated mechanisms is shown in Figure 2, adapted from [42]. Although informal, it helps to keep track of processes that could or should be exploited.

Clearly, the first issue to be decided in any mission requiring target classification is to establish which observables might be available for consideration, given the palette of operational modes of the radar and any bounds on the freedom to exploit them, as well as the physical mechanisms likely to be engaged during the observation. Then, given the prospective information content of the accessible observables, the radar can design and perform measurements tailored to yield the desired target characterization, within the limits imposed by all the contributing factors.

The extent of this being achievable is governed by the target signature. Following [41], the *generalized radar signature (GRS)* of an object *x* can be defined as:

*GRS (**x**) = response of radar when **x** is present—response of radar when **x** is absent, as recorded over the space of observables*

With this definition, it is clear that the *GRS* contains the totality of available information that can be used to characterize the object. It is equally clear that the *GRS* depends not only on the intrinsic properties of the object of interest but also the excitation actually delivered to the target zone and the transformations executed on the scattered field as it returns to the radar and passes through the radar reception and processing stages. This inescapable embedding obliges us to consider the target characterization problem in the context of the full radar observation process. As with many other HF radar tasks, this can be formulated using a radar process model as we have described elsewhere [42,43] but which we summarize here.

The process model allows us to incorporate as much or as little of the prevailing physics as may be needed for a specific application under the prevailing circumstances, to model the form in which specific interactions in the target scattering zone manifest themselves in the radar output, to optimize siting for particular missions, and to devise appropriate inversion procedures. The received signal is represented as the output of a time-ordered sequence of operators acting on the selected waveform set,

$$s=\sum_{n_{B}=1}^{N}\tilde{R}\left[\prod_{j=1}^{n_{B}}{\tilde{M}}_{S\left(j\right)}^{S\left\lfloor j+1\right\rfloor }\tilde{S}\left(j\right)\right]{\tilde{M}}_{T}^{S\left(1\right)}\tilde{T}w+\sum_{l=1}^{N_{J}}\sum_{m_{B}=1}^{M}\tilde{R}\left[\prod_{k=1}^{n_{B}}{\tilde{M}}_{S\left(k\right)}^{S\left\lfloor k+1\right\rfloor }\tilde{S}\left(k\right)\right]{\tilde{M}}_{N}^{S\left(1\right)}n_{l}+m$$

where

w represents the selected waveform set,

$\tilde{T}$ represents the transmitting complex, including amplifiers and antennas,

${\tilde{M}}_{T}^{S\left(1\right)}$ represents propagation from transmitter to the first scattering zone,

$\tilde{S}\left(j\right)$ represents all scattering processes in the j-th scattering zone,

${\tilde{M}}_{S\left(j\right)}^{S\left\lfloor j+1\right\rfloor }$ represents propagation from the j-th scattering zone to the (j + 1)-th zone,

$n_{B}$ denotes the number of scattering zones that the signal visits on a specific route from

the transmitter to the receiver,

$N_{J}$ denotes the number of external noise sources or jammers,

${\tilde{M}}_{N}^{S\left(1\right)}$ represents propagation from the i-th noise source to its first scattering zone,

$m_{B}$ denotes the number of scattering zones that the i-th noise emission visits on a specific route from its source to the receiver,

N,M denote the maximum number of zones visited by signal and external noise,

respectively,

$\tilde{R}$ represents the receiving complex, including antennas and receivers,

m represents internal noise,

s represents the signal delivered to the processing stage.

This model can be generalized to handle moving transmitters and/or receivers by implementing the frame-hopping paradigm, using Lorenz transformation operators,

$$\tilde{T}\to \;{\tilde{L}}_{T}\tilde{T}$$

and

$$\tilde{R}\;\to \;{\tilde{R}\;\tilde{L}}_{R}$$

where the frame-hopping is localized at the antennas to preserve the fidelity of signal physics representation in the propagation medium, which is almost invariably frequency dependent.

**Figure 3:**
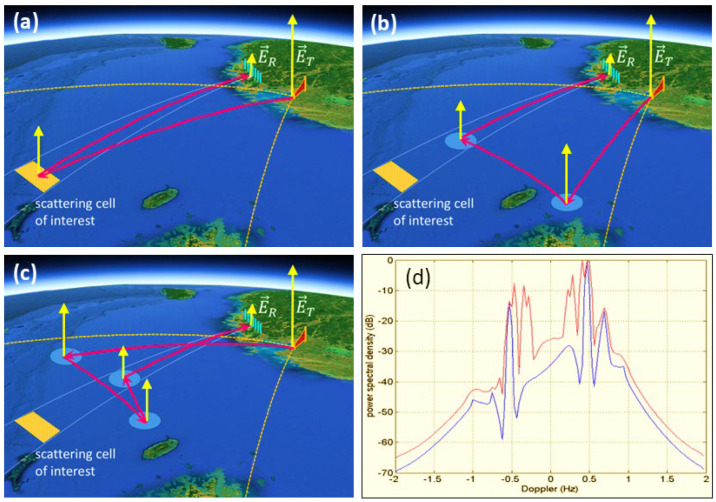
Multizone scattering processes for HFSWR (**a**–**c**), and (**d**) modeled spectra showing a corrupted spectrum for a poorly situated radar (red), with blue showing the uncorrupted spectrum.

Originally the scattering zone construct arose from the need to model multi-hop skywave propagation and range-folded echoes, but later it found application to HFSWR. The relevance in this context is illustrated in Figure 3. Here, the transmitter radiates over a broad arc, so echoes that one might naively assume all originate from the distant resolution cell of interest may in fact have multi-zone echoes superimposed, arriving from the same direction and the same group delay as those from the designated cell but possessing complex Doppler modulations due to the successive scattering processes.

The severity of the contamination is a function of the transmit beamwidth and the directional wave spectrum in the surveillance region; it can be reduced by astute site selection and the use of MIMO transmit beamforming.

Expressions for the resulting Doppler spectrum were reported in [44] and modeled in [45]. For instance, the power spectrum of a single frequency tone, after n zones, allowing only first-order scattering at each zone, takes the form described by the following expression:

$$\begin{array}{c} D_{n}\left(r_{n+1},\omega \right)\;=\; \end{array}\;\begin{array}{c} \frac{1}{{\left(64\pi \right)}^{n}}\int \ldots \int \sigma \left(\alpha_{n+1},\sigma_{n},\nu_{n};r_{n}\right)\sigma \left(\alpha_{n},\sigma_{n-1},\nu_{n-1};r_{n-1}\right)\ldots \sigma \left(\alpha_{2},\sigma_{1},\nu_{1};r_{1}\right)\\ \times {\left|F_{R}\left(\alpha_{n+1}\right)G_{0}\left(r_{n+1},r_{n}\right)G_{0}\left(r_{n},r_{n-1}\right)\ldots G_{0}\left(r_{1},r_{0}\right)F_{R}\left(\alpha_{1}\right)\right|}^{2}\\ \times \delta \left(\omega_{0}-\omega -\nu_{1}-\nu_{2}-\ldots -\nu_{n}\right)d\nu_{1}d\nu_{2}\ldots d\nu_{n}dr_{1}dr_{2}\ldots dr_{n} \end{array}$$

where $\alpha_{n}=\;\frac{r_{n}-r_{n-1}}{\left|r_{n}-r_{n-1}\right|}$, $G_{0}\left(r_{s},r_{p}\right)=\;\frac{2e^{ik\left|r_{s}-r_{p}\right|}}{\left|r_{s}-r_{p}\right|}$, $\sigma \left(\alpha_{s},\alpha_{p},\nu ;r_{p}\right)=4\pi {q_{p}}^{4}Z\left(q_{p},\nu ;r_{p}\right)\;$ is the bistatic scattering coefficient (as a function of frequency) at location $r_{p}$, where $Z\left(q_{p},\nu ;r_{p}\right)$ is the Fourier component of the surface elevation field satisfying the Bragg condition. $F_{T}\left(\alpha_{j}\right)$ and $F_{R}\left(\alpha_{j}\right)$ are the transmit and receive gains.

In most circumstances, there is no necessity to consider more than one zone because the successive contributions become progressively weaker. For commercial HFSWR systems, they would typically fall below the noise floor, though not always [46]. However, for state-of-the-art military-grade radars, that is less often the case. Such radars can achieve echo dynamic ranges (clutter-to-noise ratio (CNR), sub-clutter visibility (SCV)) exceeding 100 dB at over-the-horizon distances as a result of higher power, sophisticated electronics, and advanced signal processing.

We have previously reported modeling results that confirm the feasibility of multi-zone echo reception for skywave radar configurations [47,48], where it takes the form of side scatter in multihop signal paths. This has been observed in skywave radar experiments when land echoes from side scatter were superimposed on two-hop sea clutter along ocean paths. The relevance of this generalization to the present study emerges when we look beyond detection and consider the effects of propagation on the signal features to be employed for classification. Some target signatures are weaker than the primary skin echo, so preserving dynamic range is an imperative. Even if multi-zone echoes cannot be avoided, it may be possible to arrange for them to fall in parts of Doppler space where they do not obscure the signatures, either by siting, adaptive transmit pattern, or frequency selection.

### 3.3. A Taxonomy of Scattering Mechanisms

The simple partition of scattering mechanisms into $S_{t}$, $S_{e}$, $S_{m}$, and $S_{x}$ is useful only as a gateway to a more sophisticated categorization that extends to include statistical, syntactic, and semantic forms of target-related information that may be exploited for target characterization. The methodology we have developed over many years begins by assigning each of the various techniques we have explored into one of four classes:

1. Intrinsic: Methods in this class involve only the inherent scattering properties of targets, dependent only on shape and constitutive properties, so they are exclusively from $S_{t}$. For example, the research carried out at OSU in the 1980s falls into this class.
2. Interactive: Here, we include the signatures that result from the coupling between the target and its environment. $S_{t}$, $S_{e}$, $S_{m}$, and $S_{x}$ are all represented in this class, as follows from their definitions. Ship wakes are an obvious example.
3. Behavioral: It is reasonable to assume that the various actors in the surveillance zone are involved in goal-oriented activities, perhaps collectively, with operating parameters governed by platform design and ultimately limited by environmental conditions. Thus, there are both hard and soft constraints; an example is the choice of ship course and speed, which involves all these considerations, as well as factors such as fuel economy, travel time, and restrictions based on sailing regulations.
4. Responsive: There are occasions when a platform wishes to make its identity known to a friendly radar by means that are undetectable or at least unrecognizable by a third party. Two methods that can accomplish this have been tested and validated: IFF (‘identification friend or foe’), and maneuvers that present an agreed Doppler sequence to the radar, and impedance modulation.

**Figure 4:**
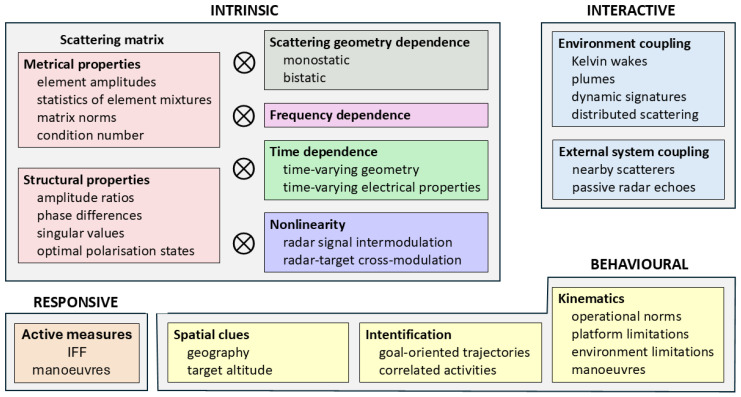
A taxonomy of the signature domains and their associated physics.

Based on these ideas, we can construct a taxonomy that breaks down the classes into sub-classes, as pictured in Figure 4. As indicated in the figure, the intrinsic methods that rely on the scattering matrix have natural extensions into the aspect (scattering geometry), frequency, time, and nonlinearity domains, opening a multiplicity of individual techniques. In the following section, we examine the phenomenology that underlies the target characterization potential of these approaches.

## 4. Signature Phenomenology

### 4.1. Intrinsic Signatures

*A.*  Time-invariant scatterers

Our primary concern in this paper is the characterization of discrete targets at far-field distances. In free space, the electromagnetic field of the radar signal is then essentially transverse, so a wave propagating in the z-direction can be written

$$\vec{E}\left(z,t\right)=\left[\begin{array}{c} E_{x}e^{i\left(kz-\omega t+\alpha \right)}\\ E_{y}e^{i\left(kz-\omega t+\alpha \right)}\\ 0 \end{array}\right]\;⟹\;e^{i\alpha }\left[\begin{array}{c} E_{x}\\ E_{y} \end{array}\right]$$

where the envelope field components $E_{x}\equiv \;E_{0}e^{i\delta_{x}}$ and $E_{y}\equiv \;E_{0}e^{i\delta_{y}}$ are complex numbers whose relative phase $\delta_{y}-\delta_{x}$ determines the polarization state of the field. The 2×1 column vector on the right is the Jones vector, a representation of the field that is well suited to following the evolution of a radar signal as it propagates, scatters, and is received by an antenna. Without compromising generality, we can select the usual H-V polarization basis for illustration.

When a radar signal scatters from a discrete target, it is often convenient to focus not on the intricacies of the currents driven in the target but on the transformation of the Jones vector of the incident field into that of the scattered field,

$${\left[\begin{array}{c} E_{x}\\ E_{y} \end{array}\right]}_{sc}=\tilde{S}{\left[\begin{array}{c} E_{x}\\ E_{y} \end{array}\right]}_{in}$$

where the operator $\tilde{S}$ has an obvious representation as a matrix $\bar{S}\;\in \;C^{2\times 2}$. This formalism is very widely used in radar, but the phenomenology is not always as simple as the equation implies. Close to the target, the scattered field is unlikely to be well modeled as a plane wave, while, further away, it may have undergone transformations in the propagation medium. This applies especially for HF radar, both skywave and surface wave, and is one of the reasons that we go to the trouble of factorizing the radar observation explicitly in the radar process model. Even so, it is extremely convenient to retain the scattering matrix construct representation, so long as we employ it within its domain of validity. The scattering matrix is the natural generator of the standard descriptions of radar scattering in terms of radar cross-section elements $\sigma_{ij}$,

$$\bar{S}=\left[\begin{array}{cc} \sqrt{\sigma_{xx}}e^{i\varphi_{xx}} & \sqrt{\sigma_{xy}}e^{i\varphi_{xy}}\\ \sqrt{\sigma_{yx}}e^{i\varphi_{yx}} & \sqrt{\sigma_{yy}}e^{i\varphi_{yy}} \end{array}\right]$$

where

$$\sigma_{jk}={lim}_{r\to \infty }4\pi r^{2}\frac{{\left|E_{sc,j}\right|}^{2}}{{\left|E_{in,k}\right|}^{2}}$$

As we shall see later, it is important to note that radar cross section thus defined leads, in many practical applications, to a second-order statistic of the scattered field and hence does not always convey all the information that could be exploited for target characterization. It is perhaps worth pointing out that there are other mathematical representations of the scattering operator $\tilde{S}$, some of which, such as the lexicographic and Pauli target vectors,

$${\vec{k}}_{L}=\left[S_{xx},\;\sqrt{2}S_{yx},\;S_{yy}\right]$$

$${\vec{k}}_{P}=\frac{1}{\sqrt{2}}\left[S_{xx}+S_{yy}\;,\;S_{xx}+S_{yy},\;2S_{yx}\right]$$

are often better suited for the mathematical operations carried out in modern signal processing algorithms. We will not pursue this here.

**Figure 5:**
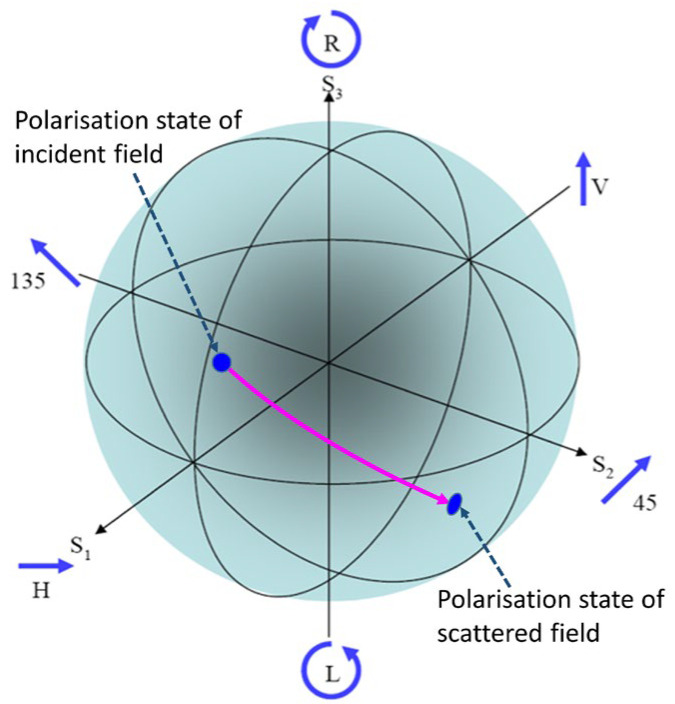
The polarization domain effect of the scattering matrix is represented by a mapping on the Poincare sphere.

In addition to any changes scattering makes to the amplitude, phase, and frequency of the incident signal, it may change the polarization state. For deterministic signals, this change has a familiar and highly useful geometric representation in the form of a mapping on the Poincare sphere, shown in Figure 5.

**Figure 6:**
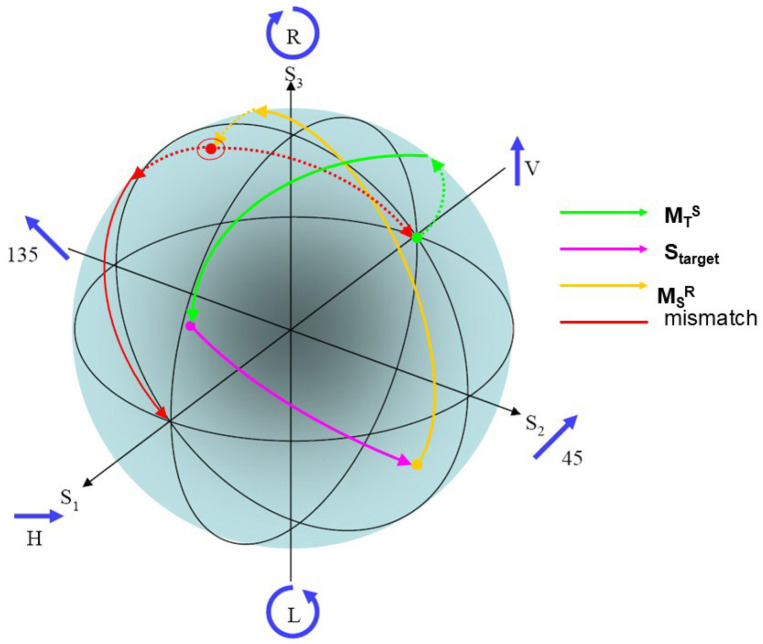
The radar process mapped on the Poincare sphere, showing the signal path from its launch at a V-polarized antenna through its sequential transformations to the reception point; we show here in red the resulting polarization mismatch to nominal V- and H-polarized receiving antennas.

The Poincare sphere is useful for more than just representing the action of the scattering matrix; it can serve as a record of the entire radar process. As marked in Figure 6, starting with the Jones vector of the field radiated from the transmitting antenna, we can model its transformation *en route* to the target by a mapping to the state of the field actually incident on the target route (the ${\tilde{M}}_{T}^{S}$ operator in the process model), followed by the action of the scattering matrix to yield the scattered field, and then the transformation experienced en route to the receiving site (the ${\tilde{M}}_{S}^{R}$ operator in the process model) where it may well fail to match the optimum polarization state of the receiving antenna. A little thought leads us to the realization that even the HF surface wave radar process cannot always be projected into the subspace of transverse magnetic or TM fields (approximately vertically polarized) without rejecting multiple scattering mechanisms that can contribute appreciably under some circumstances. Scattering from cruise missiles provides one example.

And there is another consideration. The Poincare sphere provides a full representation of the signal state space for pure states of the field, but we can draw an analogy with optics by recognizing that states corresponding to partially polarized fields can also be represented by extending the state space from the two-dimensional surface $S^{2}$ into its interior, the three-dimensional unit ball, $B^{3}$. This holds particular relevance to HF skywave radar, as experiments have shown that skywave propagation depolarizes propagating fields as well as repolarizing them. Exploiting polarization to the maximum extent in skywave radar demands proper consideration of this and the use of techniques to monitor the degree of polarization.

Keeping these considerations in mind, we can see several ways to characterize a target with a view, to discriminate it from other scatters of different form (classification), or to associate it with a known class (recognition), based only on measurements of one or more elements of its scattering matrix.

First, we may base our decision on the magnitude of a single element of the scattering matrix, as may well be the only option for radars with propagation essentially limited to a single polarization state, such as HF surface wave radar. To assess the power of this approach, we need to familiarize ourselves with representative magnitudes of the matrix elements for the classes of targets likely to be encountered. There are countless possibilities, but we can make some initial observations by considering just two different vessels and restricting our comparisons to the case of HFSWR, where the V-V element dominates.

**Figure 7:**
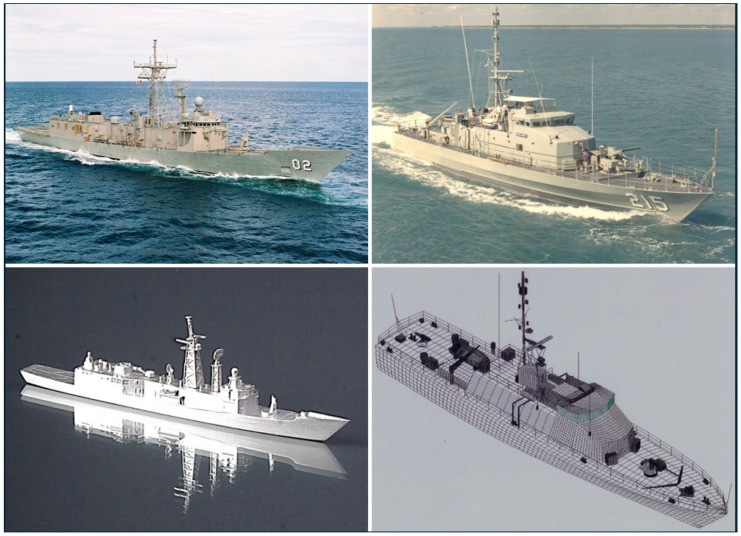
The upper left image shows the FFG 7, while that on the upper right shows the Fremantle Class Patrol Boat. The lower left image shows the scale model FFG-7 sitting on the aluminum ground plane that represents the smooth sea surface, while that on the lower right shows the digital wire model used for the NEC 4 calculations.

We have chosen the Oliver Hazard Perry FFG 7 frigate (~4000 t, 124 m) and the Fremantle Class Patrol Boat (220 t, 42 m) to represent two well-populated classes that are not ridiculously incommensurate but nevertheless might be thought to be easily distinguishable, a conjecture we will now explore. Figure 7 presents images of the two vessels, along with the sources of our scattering matrix data-scale model measurements in an anechoic chamber for the FFG and computational modeling (NEC 4) for the FCPB.

**Figure 8:**
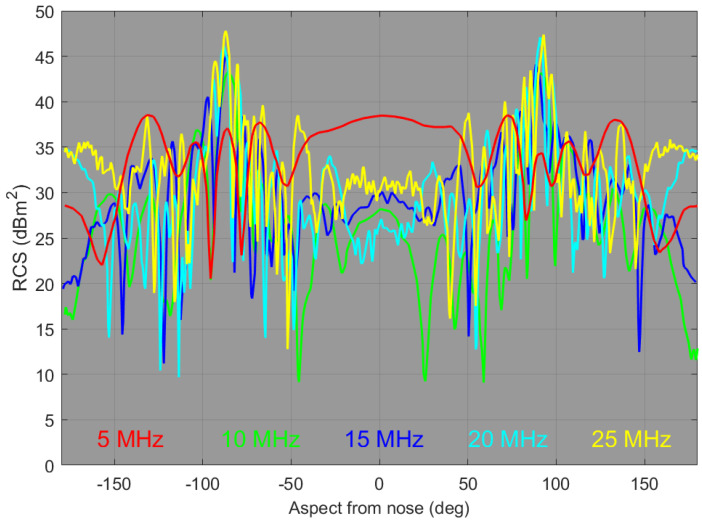
The V-V RCS element for the FFG-7 as a function of aspect for grazing incidence, at radar frequencies 5, 10, 15, 20, and 25 MHz, illustrating the difficulty of exploiting frequency dependence.

The V-V radar cross-section element for the FFG is plotted as a function of aspect in Figure 8, with curves for five frequencies overlaid.

**Figure 9:**
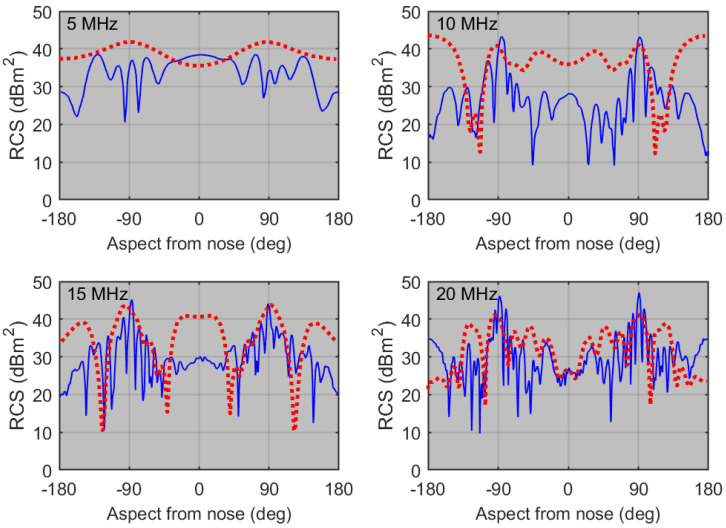
The V-V RCS element for the FFG (blue) and the FCPB (red) plotted as functions of aspect for radar frequencies of 5, 10, 15, and 20 MHz.

The point we make here is that, over most of the aspect domain, the RCS fluctuates rapidly, except at the low end of the HF band. Moreover, the frequency dependence offers little prospect for classification from the technique of ranking the responses, even if the respective propagation losses can be determined. However, it is instructive to compare the RCS elements for the two vessels, which we do in Figure 9.

**Figure 10:**
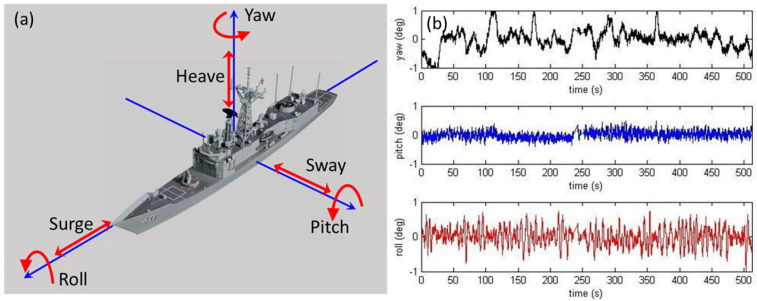
(**a**) The six degrees of freedom of a rigid ship, and (**b**) measured responses for an FFG in a low sea state. Much greater fluctuations occur in rough seas. For a frigate in the North Atlantic in winter, roll can easily exceed 15°, pitch 5°.

Here, we see a glimmer of hope for all but the highest frequency over most of the aspect domain. But there is another complication. Ships are in dynamic interaction with the ocean wave field, with each of the six degrees of freedom shown in Figure 10 subject to excitation.

**Figure 11:**
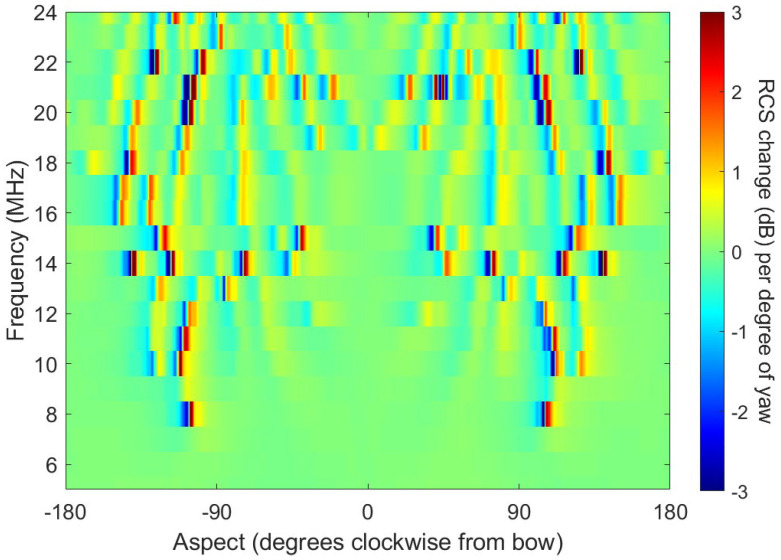
The sensitivity of the RCS element for the FCPB as the boat undergoes yaw.

We shall examine this subject in more detail later, in the context of dynamic signatures, but, for now, consider only the yaw motions. As the aspect changes, so does the RCS, and in the case of the FCPB, by up to several dB per degree of yaw at some aspects, as illustrated in Figure 11 for the FCPB, at integer frequencies from 6 to 24 MHz. To estimate the RCS for classification purposes, we need to know the scattering geometry accurately. This makes heavy demands on the target tracking subsystem, another example of the need for high connectivity in the system architecture.

Of course, the extent of the motions is a function of many variables, but, for a given ship, it is mainly dependent on heading, speed, and the directional wave spectrum. In principle, a well-designed radar can deliver all this information.

**Figure 12:**
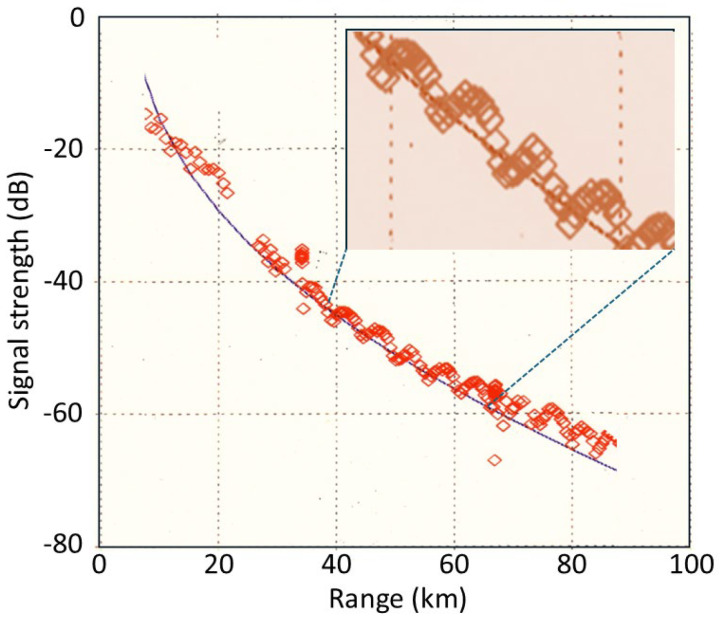
Range scalloping of the signal from a linear FMCW waveform radiated from a small boat. The black line is the predicted path loss according to the GRWAVE algorithm.

It is evident that every dB is important, so we need to look at other effects that could compromise classification. One such effect is the scalloping loss that modulates the signal as the target (or emitter) moves through range bins. Figure 12 shows an example recorded with the ILUKA HFSWR in 1997. Hamming window apodization was used for the range processing FFT during that experiment; the theoretical scalloping loss in this case is 1.78 dB, which is in agreement with the data.

**Figure 13:**
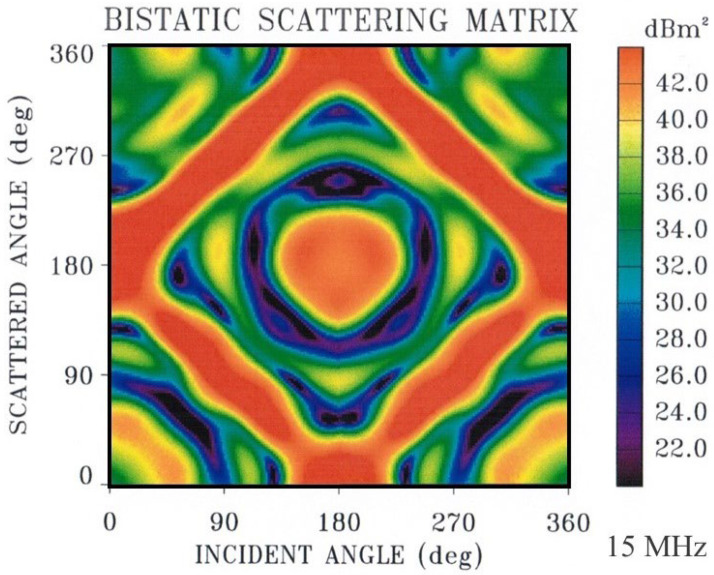
The bistatic V-V element RCS matrix for the FCPB at grazing incidence for HFSWR applications.

**Figure 14:**
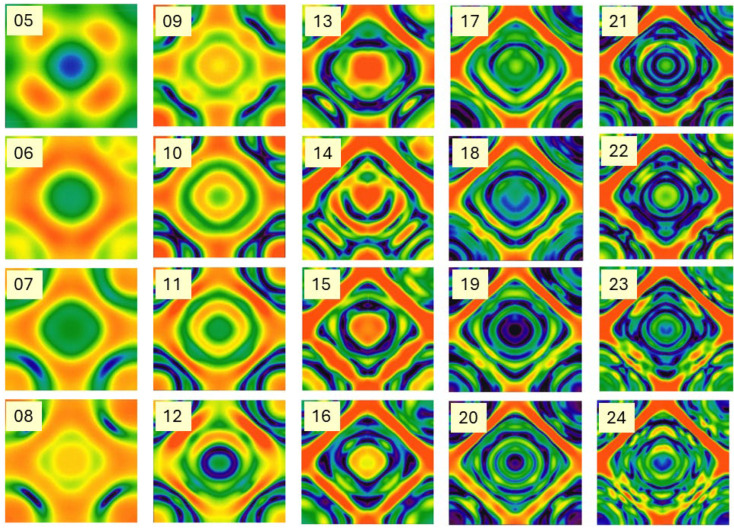
A mosaic of bistatic RCS matrices such as that in Figure 13. The frequencies (in MHz) are marked on the tiles.

So far, we have illustrated the phenomenology with model predictions and measurements from monostatic radars. Bistatic radar configurations [49] increase the complexity of target classification because the radar-target scattering geometry is constantly changing, but the increased dimensionality of signature space can also prove advantageous, as with task scheduling. Figure 13 shows an example of the bistatic V-V RCS element for the FCPB at a single frequency, while Figure 14 is a mosaic of the same element computed at frequencies from 5 to 24 MHz.

There is obvious value in examining datasets such as this when designing minimal sets of radar frequencies for efficient classification, transmitted sequentially or concurrently depending on the radar design.

**Figure 15:**
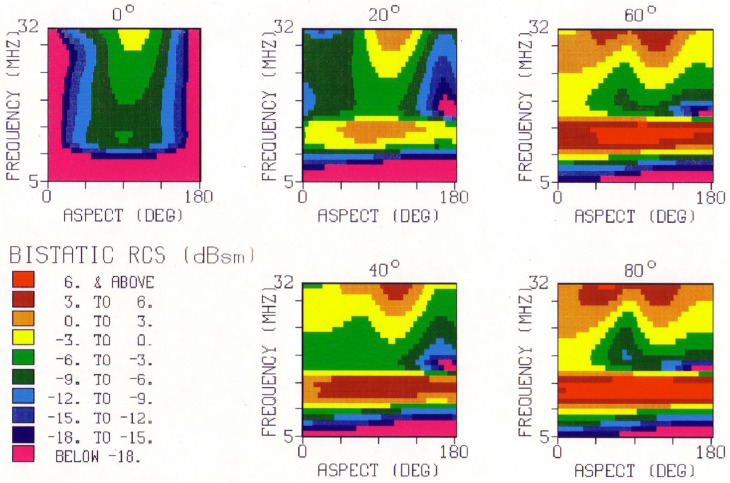
Free-space bistatic V-V RCS elements as functions of aspect and frequency, shown for bistatic angles of 0°, 20°, 40°, 60°, and 80°. Note how increasing bistatic angle increases the RCS at a band of frequencies well below that of a half-wave dipole of length equal to the aircraft vertical dimension.

HFSWR can also be tasked with detecting and classifying aircraft, providing that the three-dimensional spatial distribution of total field strength is understood and exploited. The corresponding RCS elements for a small aircraft—the AerMacchi M.B.326 (Aermacchi, Varese, Italy)—are shown in Figure 15 to illustrate the reduced RCS element magnitudes for such targets.

**Figure 16:**
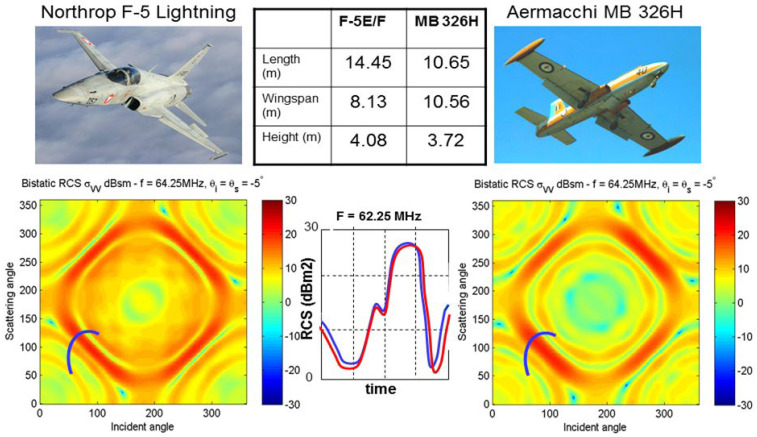
Comparison of H-H RCS element values for two aircraft following the same hypothetical flight path.

Despite the apparent richness of information in the matrix of bistatic scattering RCS, the fact that scattering from aircraft at HF falls in the resonance regime means that the general form of the matrix is common to many targets. Consider the example in Figure 16, showing the bistatic H-H RCS matrices for two aircraft—the Macchi and the F-5 Lightning. (This modeling was done at a low VHF frequency, not HF, but serves just as well.) On each panel, we have marked with a black line a hypothetical trajectory of the target in angle space as it follows some flight path. Between the panels, we plot the two RCS element histories along the path, revealing a high degree of similarity.

The lesson here is that extending the signature domain from point values to flight paths may not be sufficient.

**Figure 17:**
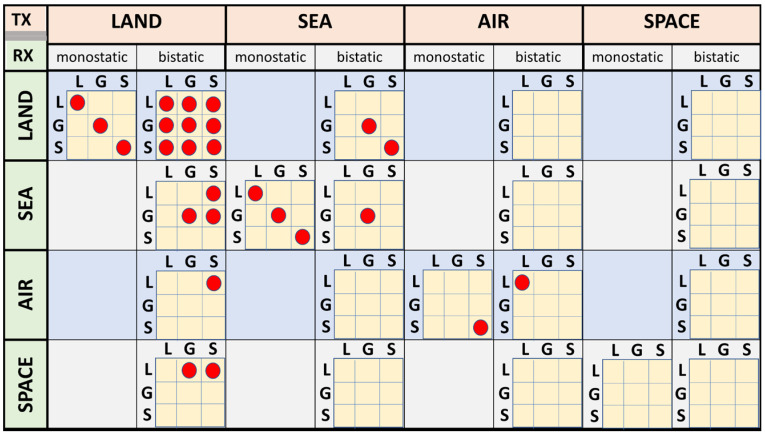
A taxonomy of HF radar configurations using line-of-sight (L), ground wave (G), or skywave (S) propagation on the signal paths to and from the target. Red dots mark some that have been reported in the literature.

We now turn attention to situations where the full scattering matrix is actively involved. This includes all configurations where skywave propagation is involved, fully polarimetric line-of-sight radars, and passive radars where the receive site has antennas able to deliver orthogonal polarization states. Figure 17, reproduced from [49], presents a comprehensive taxonomy and identifies some of the many HF radar configurations that have been implemented, at least in experiments if not in operational service.

**Figure 18:**
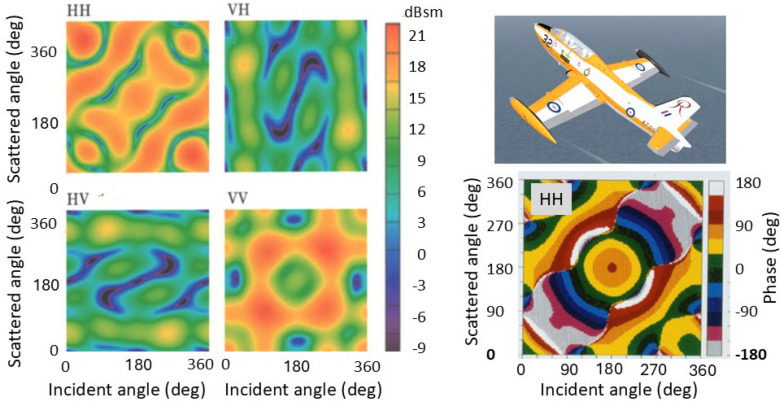
The polarization power scattering matrix of the AerMacchi MB 326H is shown on the left for an elevation angle of 10° and a radar frequency of 15 MHz. The upper right panel shows the aircraft, the lower right maps the phase of the H-H elements.

An example of the complex scattering matrix for an aircraft is presented in Figure 18. Here, the target is the AerMacchi MB 326H; the figure shows the magnitude (squared) of the elements of the polarization matrix, along with the associated phases for just one of the four elements. (The odd choice of color scale for the phase information is a legacy from its creation four decades ago [50].) The relevant point for target classification is that, for aircraft of fighter size (the AerMacchi has exactly the same wingspan as the F-35 Lightning A, though it is 30% shorter in length), the gradients in these quantities tend to be modest relative to tracking accuracy.

**Figure 19:**
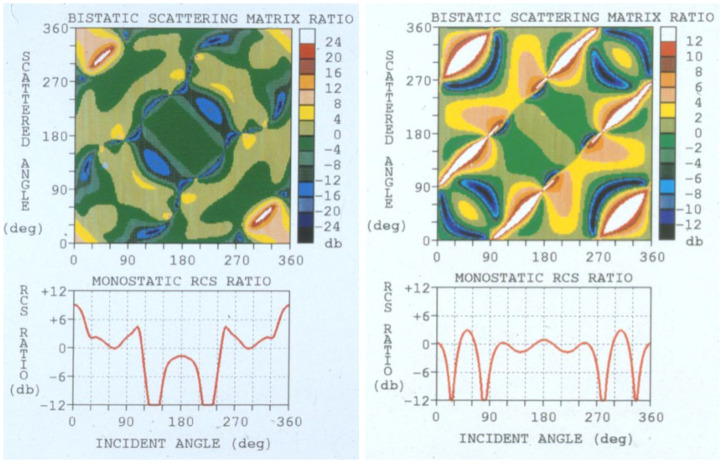
Element by element ratios (dB) for the Aermacchi relative to Aircraft Z at two frequencies. The lower traces are the monostatic RCS ratios, i.e., the trailing diagonals of the upper panels.

The most direct use of this kind of information for discriminating between two target species is to compare corresponding elements by taking the ratio. Two examples of this are shown in Figure 19, retrieved from [41]. One of the aircraft is the AerMacchi, the other is an in-service aircraft that cannot be identified here. The AerMacchi RCS is used as the numerator.

It can be seen immediately that, in the figure on the left, much of the matrix is colored green or khaki, corresponding to values in the range [−4, 4] dB. In other words, there is little discrimination power there. In contrast, at the frequency used to generate the figure on the right, a much larger fraction of bistatic geometries presents ratios exceeding [−4, 4] dB. This appears to hold much greater promise, but there is a secondary consideration: if the radar is a monostatic radar, then the ratios take the form shown in the smaller panels, below the matrices, showing the values along the trailing diagonal. Now the situation is reversed, with the frequency on the left providing nearly twice as much aspect extent above the threshold, though still less than 30%. A monostatic radar is not optimum for this assignment problem.

The appearance of randomness in the measurements, arising predominantly through the propagation operators ${\tilde{M}}_{T}^{S\left(1\right)}$, ${\tilde{M}}_{S\left(n\right)}^{R}$ and geometrical uncertainties obliges us to apply statistical techniques. We explored this long ago with a skywave radar by constructing scatterers with distinct scattering matrices and then collecting echoes under a range of ionospheric conditions. The same approach was used with many known ship targets that were tracked for long periods.

**Figure 20:**
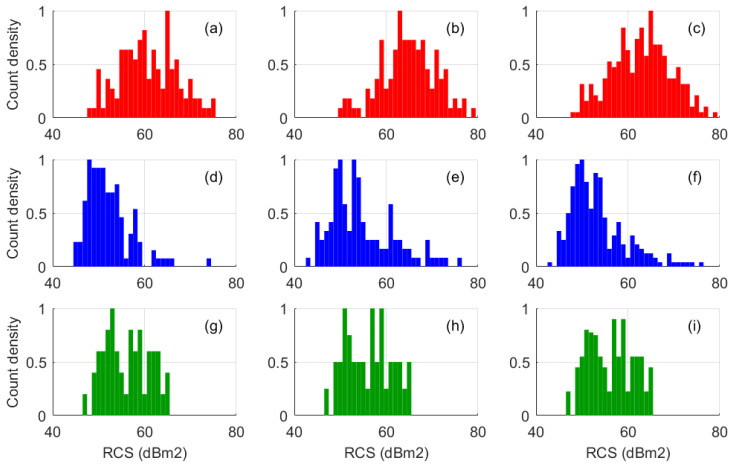
Histograms of echo strength recorded from three targets (identified by color: red, blue, green), recorded on three consecutive days (shown left to right across the page).

A crude way of inspecting the data is to present it in histogram form, as illustrated in Figure 20. The echoes from three targets (red, blue, green) were accumulated over a two-hour period on three consecutive days, that period varying from mid-morning (left column) to midday (center column) and then mid-afternoon (right column), in the expectation of a consistent response. There is a danger in adopting this approach because there is no simple way of knowing whether the sampling has been uniform over the stochastic parameter space—the orientation and ellipticity of the polarization state, say—but these histograms did reveal some reasonably consistent forms and with the incorporation of auxiliary information might prove useful. If the shape cannot be trusted, some key order statistics might prove to be more robust signatures.

**Figure 21:**
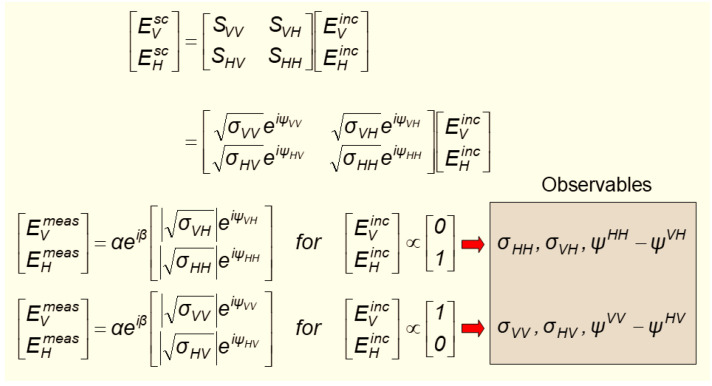
Observables at the receiver when two polarization channels and their phase difference are recorded for either polarization illuminating the target.

It may be possible to classify some targets from scalar measures of the scattering matrix according to some metric, such as the Frobenius norm or the condition number, without regard to the structural properties, but it is the latter that holds the greater promise for techniques and guidance. The simplest compound to use as a feature for input to a classifier is a pair of elements from the matrix, such as VV and HH or VV and HV. An extension of this approach is to add a third quantity-differential phase (see Figure 21).

**Figure 22:**
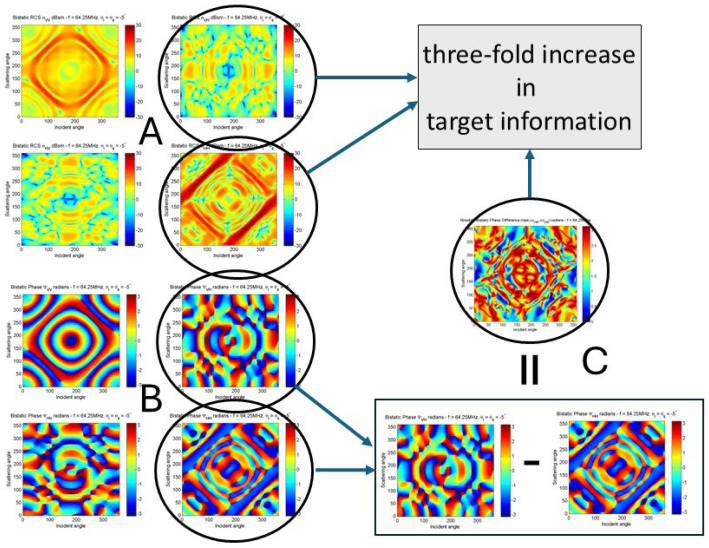
Extracting outputs from two polarization channels VH and HH, both circled in (**A**), and their differential phase Ph(VH)—Ph(HH) obtained from the circled channels in (**B**) and plotted in (**C**), expands the classification space.

We have investigated this in the context of a passive radar, where transmissions from a commercial TV station provide the illumination of opportunity. In this system the passive radar receives on both horizontal and vertical polarizations, and these are phase calibrated. The radar does not know the transmitted polarization. The targets are those shown in Figure 16. With this configuration, the number of observables increases three-fold, as shown in Figure 22, which maps the three classification subspaces.

The aim of that investigation was to assess the fraction of the bistatic scattering geometries included in the matrix for which the response differences between target types exceed a threshold and thereby provide a test statistic for classification [51].

**Figure 23:**
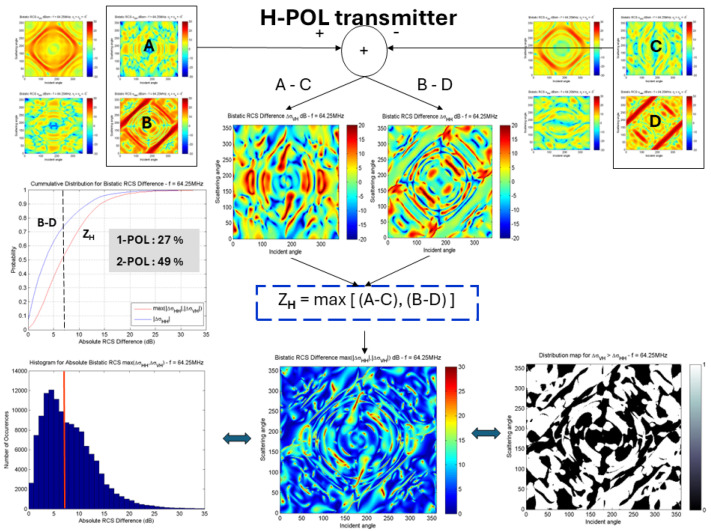
Comparing differences between corresponding polarization channel outputs for the two aircraft to find the better test statistic: H-POL transmitter case.

**Figure 24:**
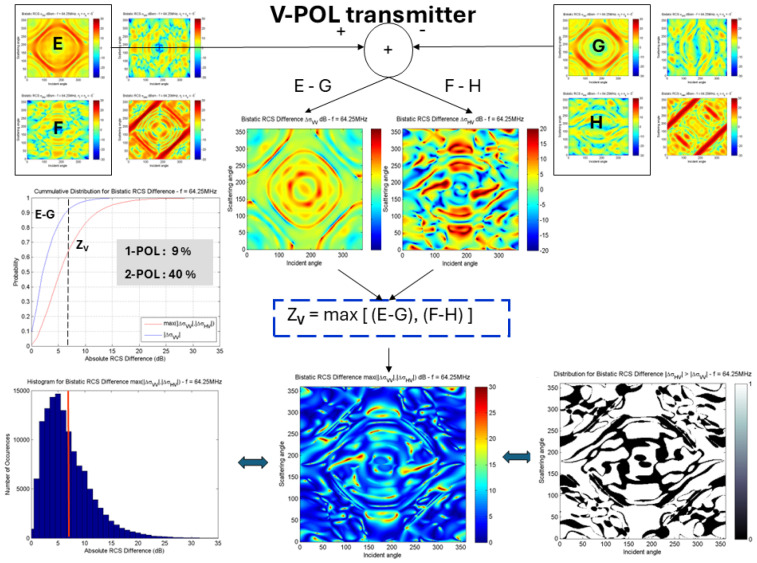
Comparing differences between corresponding polarization channel outputs for the two aircraft to find the better test statistic: V-POL transmitter case.

Figure 23 quantifies the benefits for the F-5/Macchi classification task using just the two RCS element values, when the transmitter is H-polarized, while Figure 24 does the same when the illuminating transmitter employs V polarization. In the former case, a randomly chosen single channel yields a 27% chance of exceeding the threshold averaged over elements in the bistatic difference matrix, while working from two channels achieves 49%. With the H-polarized transmitter, the corresponding values are 9% and 40%.

**Figure 25:**
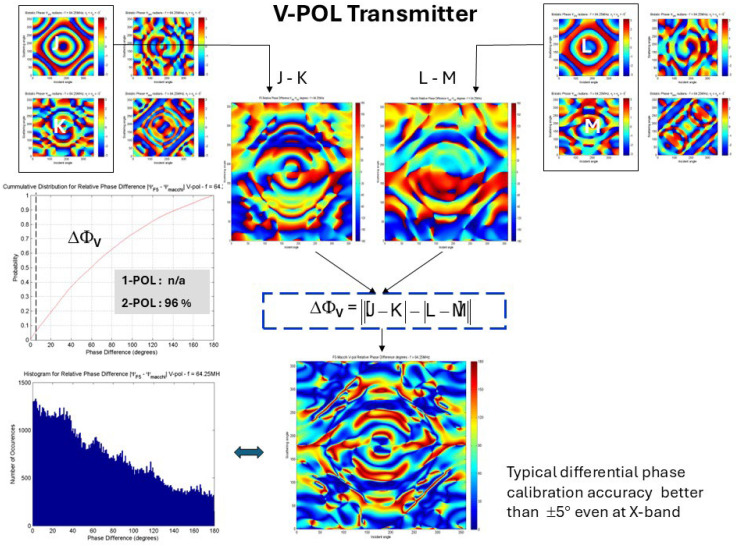
Using the phase difference between the two polarization channels (J–K for one aircraft, L–M for the other) as a test statistic is effective for 96% of the elements in the bistatic phase matrix for a V-POL transmitter.

Now we look at the use of the phase difference, bearing in mind that a measurement accuracy of ± 5° is easily achievable. Figure 25 shows the V-POL case, where 96% of cells in the bistatic phase matrix exceed this threshold, so phase difference is a powerful discriminator.

The concepts described above for the passive radar case have their counterparts with polarimetric radar, where the polarization state of the radiated waveform is under the radar’s control. With skywave radar, some subtleties arise because of polarization transformation in the propagation operators ${\tilde{M}}_{T}^{S}$ and ${\tilde{M}}_{S}^{R}$. Central to the use and success of structural techniques is full polarimetric capability and, in most cases, a facility for estimating the polarization state of the field in the target zone. This can be done using sea clutter, terrain features, known targets in the vicinity, and one or two other methods. This can be challenging, of course, and we caution that some issues remain unresolved. We shall set those issues aside and focus on the intrinsic scattering matrix attributes that have potential for exploitation.

So far, we have treated the elements of the scattering matrix as time-invariant quantities that describe the linear response of a target to an incident electromagnetic field. Many targets have time-varying geometry or time-varying electrical properties, while some manifest nonlinear behavior. It transpires that, for different reasons, these complications present particularly accessible radar signatures, as discussed below.

*B.*  Time-dependent scatterers

For scattering in the resonance regime, it is seldom meaningful to isolate the contributions to the scattered field from individual parts of the target, some of which may be moving relative to others and all of which are electrically coupled. Formally, the scattering problem involves time-varying boundary conditions disposed across the changing geometry but, for nonrelativistic targets, it may be approximated quite accurately by the expedient of computing the field scattered from a target whose spatial configuration is taken as instantaneously at rest in the coordinate frame of its center of mass (the quasi-stationary approximation [52]). In that reference frame, the frequency spectrum (‘Doppler’) of the field scattered from the target can be written

$${\vec{E}}_{scat}(\omega )=\int {\vec{E}}_{scat}(t)e^{-i\omega t}dt=\int \tilde{S}(t){\vec{E}}_{inc}(t)e^{-i\omega t}dt$$

so, for a time-harmonic incident field,

$${\vec{E}}_{inc}(t)={\vec{E}}_{0}e^{i\omega_{0}t}$$

and

$${\vec{E}}_{scat}(\omega )=\int \tilde{S}(t){\vec{E}}_{0}e^{-i(\omega -\omega_{0})t}dt$$

In the case of periodic modulation of the target geometry (or electrical properties), with some period T and corresponding fundamental frequency Ω ≡ *T*^−1^, the scattering matrix has a representation as a Fourier series,

$$\tilde{S}(t)=\sum_{k=-\infty }^{\infty }{\tilde{S}}_{k}e^{ik\Omega t}$$

Substituting,

$${\vec{E}}_{scat}(\omega )=\int \sum_{k=-\infty }^{\infty }S_{k}{\vec{E}}_{0}e^{-i(\omega -\omega_{0}-\Omega )t}dt\;=\sum_{k=-\infty }^{\infty }{\tilde{S}}_{k}{\vec{E}}_{0}{\int e}^{-i(\omega -\omega_{0}-k\Omega )t}dt\;=\sum_{k=-\infty }^{\infty }{\tilde{S}}_{k}{\vec{E}}_{0}\delta (\omega -\omega_{0}-k\Omega )$$

Hence, after demodulation to baseband at the receiver, the signature takes the form of a line spectrum at harmonics of the fundamental frequency of modulation Ω, shifted by the common Doppler shift associated with the component of the velocity of platform along the axis bisecting the scattering angle.

This kind of signature has been observed by HF radars for at least four target classes: helicopters, propeller-driven aircraft, ships with rotating antennas, and wind turbines. Historically, helicopter line spectra were the first signatures that were automatically extracted from skywave radar echoes and compared with libraries of scatterers with known periodicities.

1. Helicopters

**Figure 26:**
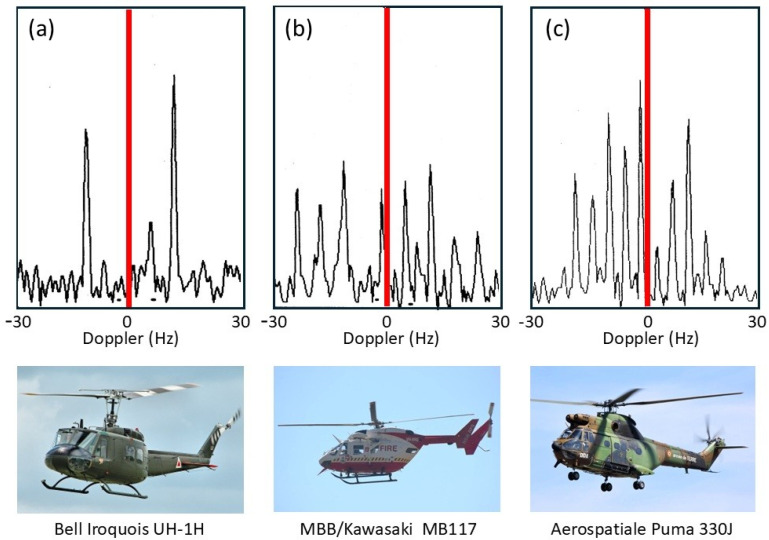
Doppler signatures of three helicopters. The red stripes mask the ground clutter which is 40–50 dB stronger than the target echoes.

In the case of a helicopter rotor,

Ω=(shaft rate)×(number of blades)

so, the line spacing alone seemingly provides a characteristic signature, unique in almost all cases and independent of the radar frequency, the line intensities, the transmitting and receiving antenna polarizations, and even the bistatic scattering angle for arbitrary geometries. Figure 26 shows the modulation signatures of three helicopters measured in 1983 with the Jindalee radar [53].

The discrimination power of these signatures is obvious, so helicopter classification/recognition is a viable mission for HF skywave radar, and suitable signal processing techniques that detect families of harmonically related spectral lines have long been implemented in operational skywave radars [54].

**Figure 27:**
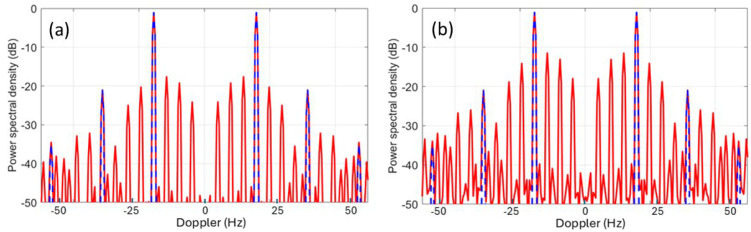
Modeled Doppler spectra from a rotating rotor structure. The blue dashed lines are what results from electrically identical blades; the red lines show two examples of what results when one blade is electrically dissimilar. In panel (**a**), the dissimilarity is 1 dB; in (**b**), it is 3 dB.

Despite its utility, this rather simple model fails to account for several features of helicopter rotor systems. Most dramatically, the assumption that the blades are electrically identical does not always hold. Helicopters occasionally need to replace individual blades with ones from later production lines, or blades sustain damage, which can be sufficient to change electrical characteristics and thereby reduce the order of rotational symmetry. The measured spectrum of the Aerospatiale SA 330J Puma aircraft shown in the third panel of Figure 26 is an instance of this. According to the simple theory, the lines should appear at a spacing of Ω = 265 *rpm* × 4 *blades* = 17.6 *Hz*. Instead, lines appear at a spacing of ~4.3 Hz in the figure. To investigate this phenomenon, we began by noting that the main rotor blades of the Aérospatiale SA 330J Puma are of composite construction, specifically glass and carbon fiber with a honeycomb core and a stainless-steel leading edge along most of the length but stopping short of the hub. We modeled the rotor blades as simple conducting rods loaded with an impedance at the hub, first for the electrically identical case and then with one blade loaded with a different impedance. The results for two settings of the impedance are shown in Figure 27, with the electrically identical case superimposed, shown by blue dashed lines. As expected, the results support the hypothesis. Clearly, by adjusting the anomalous impedance, the model spectrum could be made to match a given measured spectrum, thereby promising a means of identifying the specific helicopter, not just recognizing its type.

**Figure 28:**
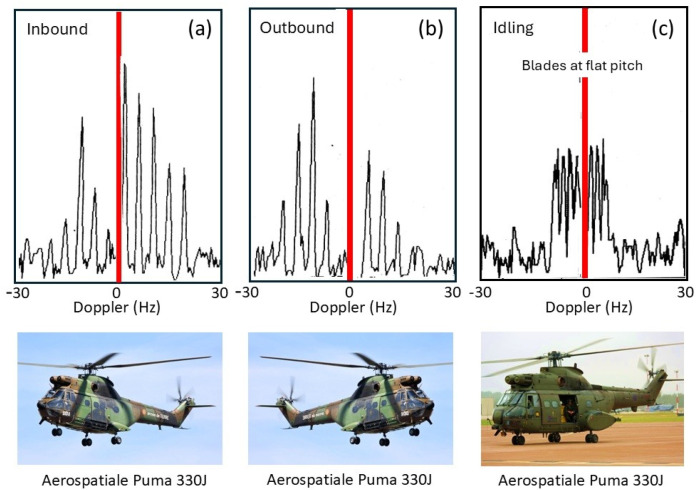
Aerospatiale Puma 330J helicopter: (**a**) Heading inbound towards radar, (**b**) Heading outbound from radar, (**c**) Stationary on ground, idling before take-off with blades flat.

Another feature of echoes from helicopters is the asymmetry in line intensities arising from the aspect presented to the radar. Figure 28 shows typical spectra for (a) a receding and (b) an approaching helicopter. This observation provides an unforeseen benefit—the ability to estimate the orientation of a helicopter from a single radar dwell, which is often impossible when the ‘DC’ Fourier component is buried in clutter. It is worth noting that the feathering of blades as a function of angle is one possible contributor to this asymmetry. Evidence to support this hypothesis can be seen in Figure 28c, which shows the Doppler spectrum of the SA Puma 330J idling while sitting on the ground before take-off. The rotation rate is low, so the spectrum is compressed, with blades flat, so the feathering asymmetry is absent. This is consistent with the data, where positive and negative components are of equal magnitude.

Yet another complication is the appearance of composite spectra arising from the additional modulation arising from the tail rotor, though this is expected to be seen only in high-dynamic-range echoes.

For HFSWR systems, where the TM electric field has less projection onto the plane of the main rotor, it might be expected that modulation from the tail rotor should dominate, but this is not observed. There are several explanations for this. First, the tail rotor has a diameter of only 3 m compared with 15 m for the main rotor. Second, the rotor–fuselage composite is the scatterer, not just the rotor, and the electrical coupling between the subsystems is the source of modulation. Third, helicopters in flight tilt the main rotor plane by means of a swash plate and employ cyclic pitch control of the individual blades, in addition to tilting the fuselage forwards when accelerating or at speed. Thus, the electric field is not orthogonal to the main rotor plane. A fourth consideration is the fact that over a finitely conducting surface, the electric field of the TM wave has a forward tilt; this is only of the order of one degree over seawater but can exceed 15 degrees over lossy ground.

2. Propeller-driven fixed wing aircraft

**Figure 29:**
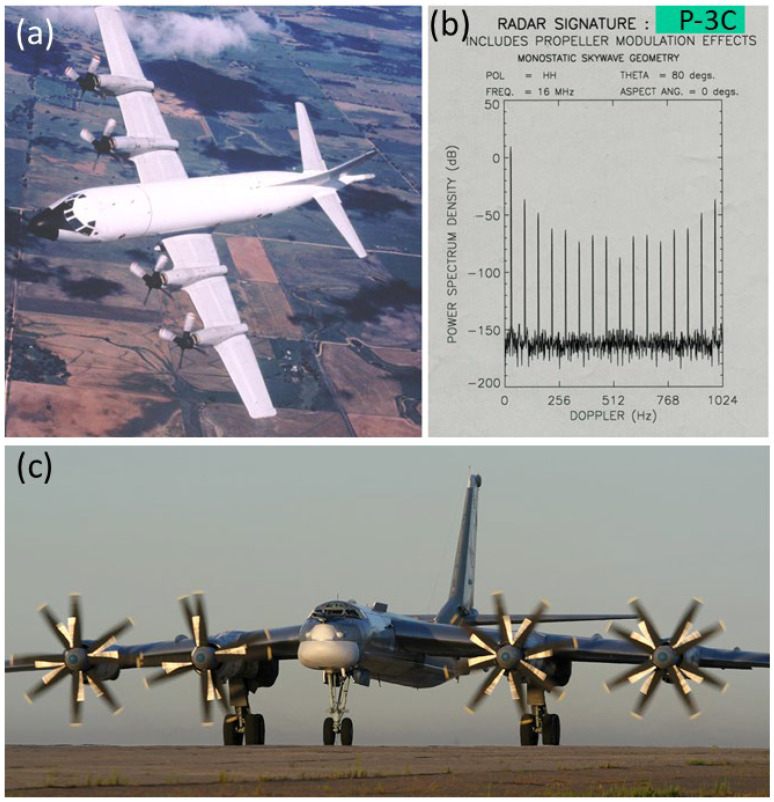
(**a**) The P3-C Orion aircraft; (**b**) a modeled spectrum from the entire body showing the contribution from the changing global geometry, dominated by the propeller motions; (**c**) the Tu-95 ‘Bear’ with its dual in-line counter-rotating propellers.

We have previously reported modeled Doppler spectra for the P-3C Orion maritime patrol aircraft (Figure 29a) with its four 4-bladed propellers each with a diameter of 4.1 m [41]. The results (Figure 29b) indicated that the strongest modulation lines would fall some 35 dB below the ‘DC’ echo, too weak to detect under a lot of propagation conditions. Nevertheless, in the 1980s, US researchers reported detections of the modulation lines of the Russian Tu-95 bomber (Figure 29c) with its four 8-bladed, counter-rotating propellers each with a diameter of 5.6 m (corresponding to roughly 3.5 dB in gain over the Orion) and rotating at 750 rpm. One might expect the fundamental frequency to approximate the sum of the clockwise and anticlockwise rates, multiplied by four, but we have not modeled this. With far greater sensitivity nowadays, propeller modulation spectra should be detectable much of the time.

3. Ship-borne antennas

**Figure 30:**
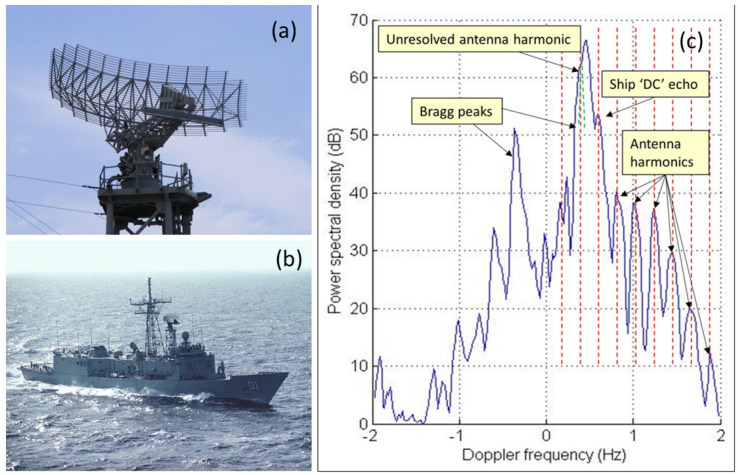
(**a**) The AN/SPS-49 radar antenna fitted to (**b**) the Oliver Hazard Perry Class FFG-7 frigate; (**c**) an example of an HF skywave radar detection of an FFG with its antenna rotating, recorded in 1984.

Although most modern warships employ fixed, phased array radars, large rotating microwave antennas were common until recently and remain in operational use in some vessels. Figure 30a shows the AN/SPS-49 antenna fitted to the Oliver Hazard Perry Class FFG-7 frigate (Figure 30b); it has a diameter of 7.3 m and has rotation rate options of 6 and 12 rpm. Figure 30c shows an example of a detection of an FFG-7 with its antenna modulation lines clearly visible. Its rotation rate is selectable from 6 and 12 rpm; for comparison, one foreign equivalent has rates of 7.5 and 15 rpm, easily distinguishable from the AN/SPS-49.

4. Wind turbines

**Figure 31:**
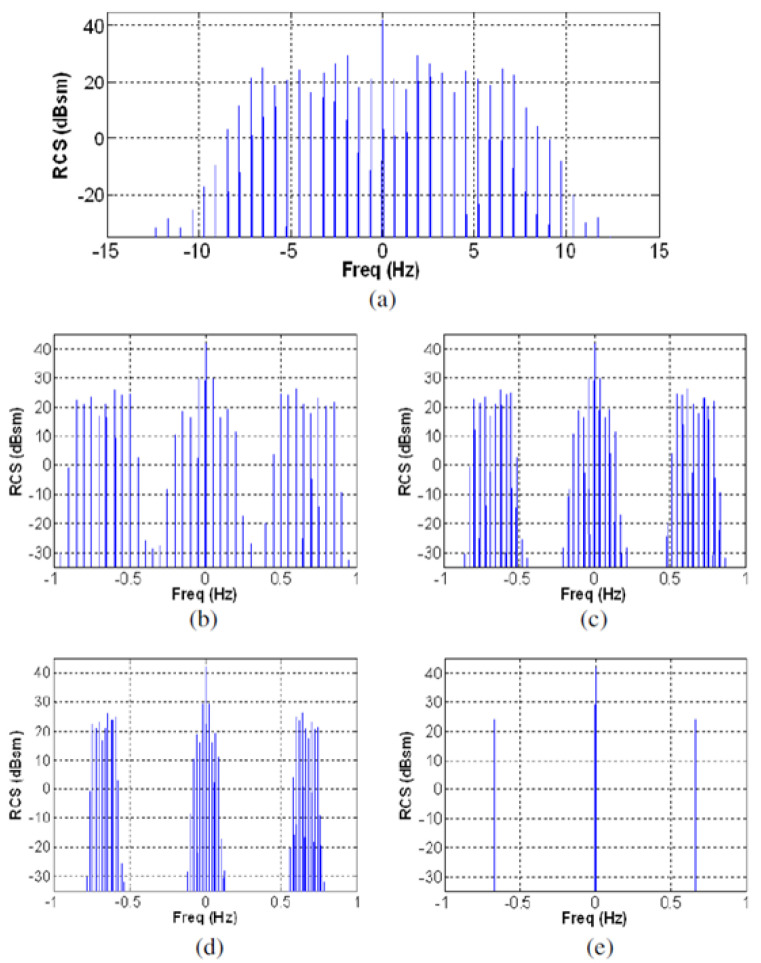
Modulation of a 13 MHz CW tone scattered by a wind turbine, showing Doppler aliasing control by varying the ratio of sampling frequency to rotation rate [56]. (**a**) 13 rpm sampled at 30 Hz; (**b**) 13 rpm sampled at 2 Hz; (**c**) 13.1 rpm sampled at 2 Hz; (**d**) 13.2 rpm sampled at 2 Hz; (**e**) 13.333 rpm sampled at 2 Hz.

HF radar echoes from wind turbines can spread across the Doppler spectrum and mask target echoes and degrade remote sensing products, so a lot of attention has been paid to characterizing them and devising ameliorative signal processing algorithms [55]. They can serve a constructive purpose for skywave radars, providing echoes from known locations and thereby providing an additional coordinate registration method. An important property of the echo spectrum—the signature—is the dependence on radar waveform. Figure 31 shows modeled spectra from a turbine illuminated with a continuous wave (CW) waveform at various combinations of sampling frequency and turbine rotation rate Ω [56].

For a sampling frequency of 30 Hz, the line spectrum is unaliased, spreading to roughly ± 12 Hz, shown in Figure 31a. At a sampling frequency of 2 Hz, as used by the CODAR SeaSonde radar, the echo is severely under-sampled, so lines beyond the fundamental are aliased and appear at spurious locations across the spectrum. By the artifice of varying *Ω* for fixed SeaSonde sampling rate, the lines can be made to appear as sidebands localized to different regions around the fundamental tones (Figure 31b–e), where the last example has the aliased lines exactly superimposed on the fundamental. If an FMCW waveform had been used, the Doppler frequency aliases would be similar, but the lines would fold into different range bins.

It is obviously important to keep this effect in mind when conducting target classification missions. Equally, one should design one’s radar with flexibility to employ a variety of waveforms that, together, are able to distinguish between different sources of modulation. The resemblance of the spectrum shown in Figure 31 to the signature of the SA-Puma helicopter discussed earlier is a case in point.

5. RADAM—RAdar Detection of Agitated Metals

Studies in the US during the late 1970s, at the Rome Air Development Center and at SRI, [57] established that the intermittent changes in electrical contacts between structural components on platforms undergoing vibration were detectable at VHF, typically 10–30 dB below the skin echo. In the case of ships in rough seas, where the hull deforms substantially, one might expect this impulse-like impedance modulation mechanism to operate under some sailing conditions, such as slamming in head seas. It may be a relatively minor echo feature, but no comprehensive implementation of a target characterization scheme should discount the possibility.

**Figure 32:**
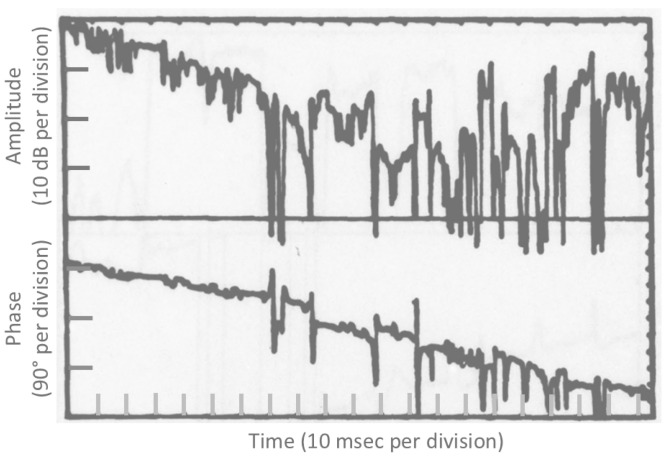
Time domain record of the VHF radar echo from an APC [57].

Unlike most of the established target signature mechanisms, it is probably detectable only in the time domain, after localization of the target echo in the frequency domain and inverse transforming. Figure 32, redrawn from [57], shows a time domain segment of the measurements from an armed personnel carrier (APC).

In most cases, the physical basis of the modulation is mechanical articulation of metal-oxide junctions, whose static electrical nonlinearity provides a different class of radar signatures, as discussed in the following section. It may be a relatively minor echo feature, but no implementation of a target characterization scheme should discount the possibility.

6. Switched impedance antennas

**Figure 33:**
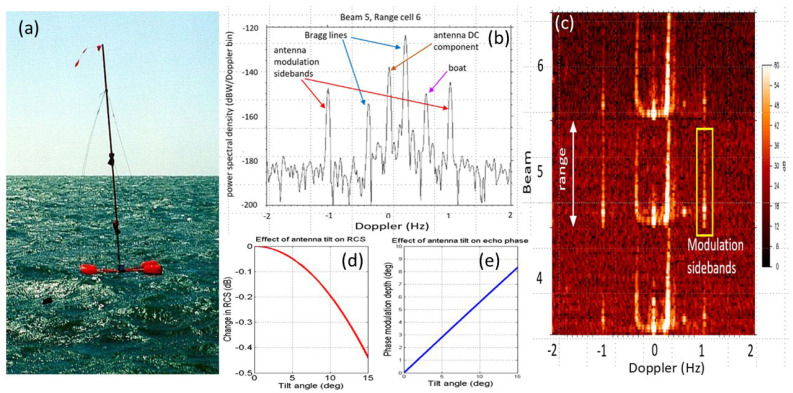
(**a**) A switched impedance buoy, (**b**) the Doppler spectrum it presents in a single range bin, (**c**) the signature distributed across a range–Doppler map, and (**d**) plots showing the sensitivity of RCS and phase to tilt.

A common utility in HF radar experiments is the switched impedance antenna, usually a monopole. An example is pictured in Figure 33, mounted on a buoy, along with its Doppler signature. In that example, the waveform repetition frequency was set equal to the antenna switching frequency so that all the sidebands fell in the same Doppler bin, spread across a number of range cells. With simple ON–OFF switching, typically half the energy remains in the DC term; with phase switching, nearly all the energy can be deposited in the Doppler sidebands. These simple implementations are useful as RCS calibration sources, for IFF, and for special propagation measurements impossible by normal means [58]. Tests using monopole lengths up to 7 m were carried out as part of the Iluka radar program, validating the theoretical models. Air-dropped versions have been developed; some were used in the 1980s.

*C.*  Nonlinear scatterers

(i) Passive IMD

Almost without exception, the dominant contributions to radar echo energy can be modeled to high precision using the assumption of linear electromagnetic response. Yet one feature of nonlinear scattering is the redistribution of energy across the frequency domain to bands free of the linear returns. When these weak echoes are detectable, they can be unique ‘fingerprints’ of the specific target involved. Moreover, radar scattering from the ocean surface is electrically linear to an extremely high degree, so the nonlinear target echoes do not have to compete with sea clutter, typically 30–70 dB stronger than the primary ship echoes.

Ships are particularly vulnerable to the generation of nonlinear products in passive structures. This can arise from physico-chemical processes of metal surface treatment during fabrication, oxidation from exposure to the marine environment (often known as the ‘rusty bolt’ effect), or the presence of foreign impurities. Measurements associated with shipboard HF communications systems have identified a host of passive contributors, including mooring or anchor chains, expansion joints, cables, slap-down plates, pipe and bracket joints, door hinges, life raft hangers, ladders, armored cables, LSO nets, antenna guying wires, guard rails, booms, gang planks, and roller curtain doors. To give an idea of the severity of the nonlinearity, one test revealed high levels of inter-modulation distortion (IMD) up to the 21st order, with measurable levels up to the 51st order [59].

In addition to passive contributors, nonlinear contamination can be traced to active contributors, especially electronic subsystems based on semiconductor components such as transmitters, receivers, navigation equipment, loudspeakers, and even deliberately nonlinear elements used to protect sensitive receivers from jamming and EMP. Coupling may occur via antennas, cables, and wires used for normal signal transmission, conducting casings, and capacitive effects.

An important point made in [60] is that scattering from semiconductors generates both even- and odd-numbered nonlinear products, whereas ‘rusty bolt’ sources produce predominantly odd orders. This gives us another tool for classification. We should also remember that IMD can be regarded as the linear radar waveform mixing with itself. There is no reason why it could not mix with other signals incident on the ship, or generated there, with much higher power densities and resulting IMD products.

The general formulation of nonlinear processes is based on the Volterra series expansion. In the radar context, a reduced description applicable to memoryless nonlinearity has been used to establish a direct mapping between low orders of nonlinear transfer functions and the corresponding orders of radar cross section [61,62]. The frequency domain generalizes to a multi-frequency domain, with the RCS elements readily interpreted as the echoing responses of the target to specific combinations of incident frequencies, highlighted in the corresponding radar equations:


$$\begin{array}{c} P_{R}\left(f\right)=\frac{P_{T}\left(f\right)G_{T}\left(f\right)G_{R}\left(f\right)\lambda^{2}}{\left(4\pi R_{T}^{2}\right)\left(4\pi R_{R}^{2}\right)4\pi }\sigma_{1}\left(f\right)+\\ +\frac{P_{T_{1}}\left(f_{1}\right)G_{T_{1}}\left(f_{1}\right)P_{T_{2}}\left(f_{2}\right)G_{T_{2}}\left(f_{2}\right)G_{R}\left(f\right)\lambda^{2}}{\left(4\pi R_{T_{1}}^{2}\right)\left(4\pi R_{T_{2}}^{2}\right)\left(4\pi R_{R}^{2}\right)4\pi }\delta \left(f-f_{1}-f_{2}\right)\sigma_{2}\left(f_{1},f_{2}\right)df_{1}df_{2}+\\ +\frac{P_{T_{1}}\left(f_{1}\right)G_{T_{1}}\left(f_{1}\right)P_{T_{2}}\left(f_{2}\right)G_{T_{2}}\left(f_{2}\right)P_{T_{3}}\left(f_{3}\right)G_{T_{3}}\left(f_{3}\right)G_{R}\left(f\right)\lambda^{2}}{\left(4\pi R_{T_{1}}^{2}\right)\left(4\pi R_{T_{2}}^{2}\right)\left(4\pi R_{T_{3}}^{2}\right)\left(4\pi R_{R}^{2}\right)4\pi }\delta \left(f-f_{1}-f_{2}-f_{3}\right)\\ \sigma_{3}\left(f_{1},f_{2},f_{3}\right)df_{1}df_{2}df_{3}\;+\;... \end{array}$$

How far we might progress through the hierarchy classification—recognition—identification depends on the extent of our prior knowledge of the target’s nonlinear RCS characteristics. Detailed knowledge is unlikely to be available in the great majority of cases, but one might well expect the strength of nonlinearity from corroded structures to increase with the age of the ship.

We have previously calculated the relative contributions of the different RCS orders on target detectability for both skywave and surface wave HF radars, using an equivalent circuit to model the nonlinearity [63]. The radar parameters (power, antenna designs, etc.) were copied from some existing radars, but the nonlinear coefficients were pure guesswork. The conclusions of that study were marginally encouraging for HFSWR but bleak for skywave radar, largely because the power law for the geometric loss in a monostatic radar takes the form *R^−(2n+2)^* for *n*-th order products, and skywave radar ranges are so extreme.

**Figure 34:**
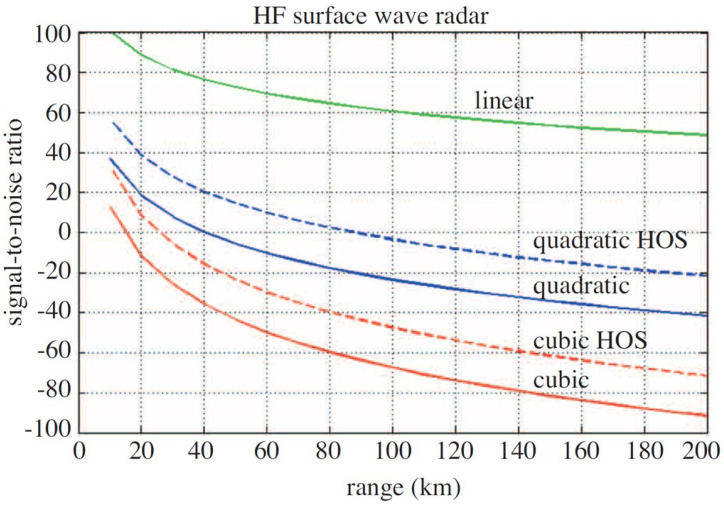
Modeled signal-to-noise ratio vs. range for different orders of nonlinearity, processing, and waveforms.

The prediction for HFSWR in a representative scenario, using classical processing of a linear FMCW waveform, are reproduced in Figure 34, where the quadratic nonlinearity coefficient was set to 10^−3^ and the cubic to 10^−2^. Note that these are voltages; the powers are −60 dB and −40 dB, which we suspect are very conservative values.

There are several measures that can be adopted to improve the prognosis, some of which were considered in [63,64]. First, use of higher-order statistical processing yields a gain in SNR when the noise background is Gaussian. For example, modeling showed an achievable gain of nearly 20 dB for third-order IMD based on sensible record lengths, as shown in Figure 34. Second, instead of using a continuous waveform, suppose we could employ a pulse waveform with a duty cycle α. For the same average transmitting power, this enhances the strength of the IMD by a factor of *α^−n/2^*. Once detection has been achieved, one might trade off some of the range footprint to permit a lower duty cycle, 25%, say, or even 10%. The former yields an enhancement of the third-order IMD by 9 dB, the latter by 15 dB. Even for second-order products, the corresponding gains are 6 dB and 10 dB. Were the transmit power to be increased to that used in some military-grade radars, the second-order IMD would have an SNR exceeding 5 dB at a range of 200 km.

We have not looked at the exploitation of phase information, discarded in the power spectrum but retained in the higher-order spectra. Whether this could have potential for target characterization is an open question.

(ii) Active IMD

**Figure 35:**
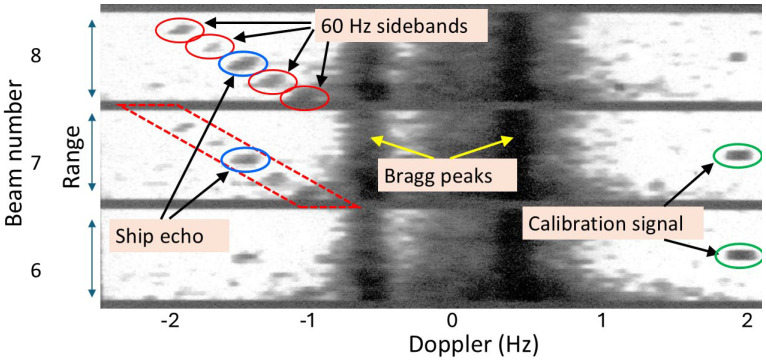
Part of an azimuth–range–Doppler display showing a ship echo and 50 Hz sidebands. The sidebands are indicated collectively in beam 7 and individually in beam 8.

Skywave radars have, on occasion, observed another form of nonlinear signature whose origin remains a matter of speculation, though with some evidential support from the operational context. A frame from one of those instances is presented in Figure 35, which shows a range–Doppler map with a family of harmonically related lines, spaced by 60 Hz and hence heavily range-aliased. (The individual range lines here are actually multiple range bins averaged noncoherently.)

Our hypothesis starts from the fact that scattering from antennas involves two mechanisms—the structural mode and the antenna mode. The former comprises all the sources that reradiate when a matched load is connected to the antenna feed port, and there is no reflected power from the load, while the latter refers to the contribution from power that is reflected from the load and reradiated into space. If the load were excited with an internally generated field, that modulation could be transferred to the external signal that is being reflected from the load and reradiated. One likely source for such an unwanted internal field is mains power.

The context of the observations was as follows. The skywave radar which acquired the signature in Figure 35 was involved in a nocturnal exercise that would have drawn the attention of any country interested in this technology. A discreet form of monitoring radar transmissions in the 1- propagation zone, along with echoes from any targets being illuminated, is to position a vessel in the radar footprint and listen, with an HF antenna feeding a sensitive receiver. Given shipboard constraints, mains leakage into the system might be unavoidable and dealt with further through the processing chain. Most ships employ either 50 Hz or 60 Hz mains frequency to power electronic systems, depending on country of origin, so measuring the modulation frequency narrows the options.

*D.*  Structural properties of the scattering matrix

Analysis of the scattering matrix can proceed in a number of ways. For example, it may be written as the sum of two or more matrices, each of which is associated with a specific mechanism, or it may be factorized and written as the product of two (or more) matrices, representing sequential scattering processes, or one might find its eigenfunctions, which could identify useful probing states. There are other possibilities, and each approach has its applications. Two approaches have been explored within the HF radar community: optimal polarization states and characteristic modes.

(i) Optimal polarization states

Given a matrix, one of the first steps one might take to explore its physical significance is to find its eigenvectors. In the radar context, there is a complication that must first be addressed—the need to adopt a polarization convention for the radar scattering process—either the Forward Scattering Alignment (FSA) or the BackScatter Alignment (BSA). We automatically embed that dichotomy in our radar process model but, when dealing with explicit representations, some care must be taken. The standard formulation for the scattering matrix—the Sinclair matrix—uses the BSA, while most optical processes are framed in the FSA with Jones matrices replacing the Sinclair matrix. The eigenvalue problem in the BSA becomes a coneigenvalue problem,

$$\tilde{S}\vec{x}=n{\vec{x}}^{*}$$

where ${\vec{x}}^{*}$ denotes the complex conjugate to $\vec{x}$; the FSA is conventional.

We can address our target characterization problem in either convention by focusing instead on the received power, represented by the Graves power scattering matrix, $\tilde{G}=\;{\tilde{S}}^{H}\tilde{S}$, satisfying

$$\tilde{G}\vec{x}=m\vec{x}$$

with $m=\;{\left|n\right|}^{2}$. The optimal eigenvectors fall into five categories:

- the co-polarization maxima (CO-POL MAX)
- the co-polarization nulls (CO-POL NULL)
- the cross-polarization maxima (X-POL MAX)
- the cross-polarization nulls (X-POL NULL)
- the cross-polarization saddle points (X-POL SADDLE)

**Figure 36:**
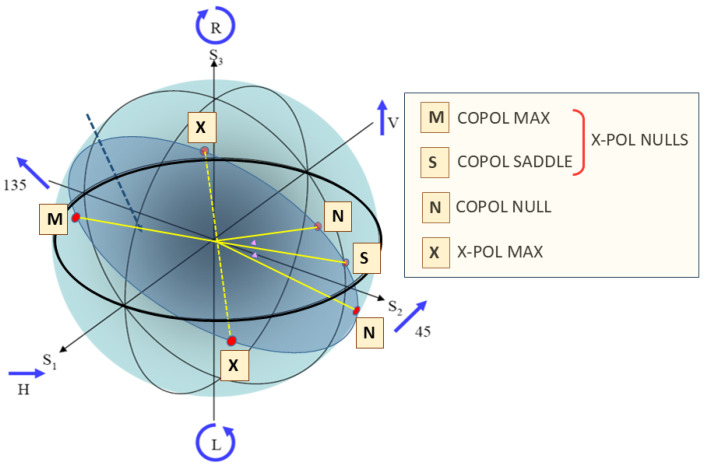
The Poincare sphere with the Huynen fork states identified.

In the case of a symmetric scattering matrix representing monostatic measurements in a reciprocal propagation medium, the eigenvectors of $\tilde{G}$ are also the eigenvectors of $\tilde{S}$; moreover, the CO-POL MAX states coincide with the X-POL NULL states. It was shown by Huynen [65] that these states have a geometric interpretation as a fork configuration on the Poincare sphere, pictured in Figure 36.

**Figure 37:**
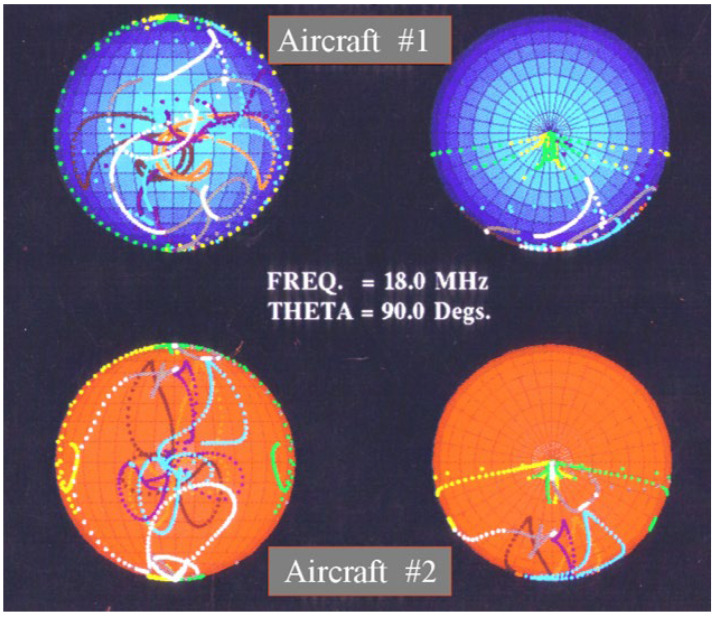
Examples of the trajectories traced out on the Poincare sphere by the optimal states for two in-service military aircraft as they execute a sweeping turn [41]. The colors represent different optimal polarization states; the figures on the left view from latitude 0°, longitude 0°, those on the right from the North pole at latitude 90°.

The optimal polarization states characterize the scattering matrix completely, with the advantage that, unlike the scattering matrix or the Stokes parameters, the description is independent of the polarization basis. Any changes to the scattering properties of the target are reflected in the dynamics of the optimal polarizations. Systematic variations of the S-matrix properties map into eigenvector trajectories on the Poincare sphere, as illustrated in Figure 37.

A strategy for target classification could take the form of sampling with different transmitted polarizations to find one or other NULL state, as nulls are generally sharper than maxima and hence yield more accurate estimates. Recognition would rely on a pre-computed library.

(ii) Characteristic modes

The scattering matrix describes asymptotic properties of the field scattered by the target, not the response elicited in the target itself. It was pointed out by Garbacz [66] that, for targets in the resonance regime for scattering, that is, with dimensions in the range 10^−1^–10^1^ wavelengths, say, the natural eigenstates of the current distribution induced on the target, should form a meaningful basis for describing the target’s scattering behavior. Moreover, in most circumstances, only a few eigenstates are likely to dominate, much like the dipole and quadrupole moments in Mie scattering.

Central to the utility of characteristic modes is the fact that they are inherent properties of the target, independent of any incident field. What changes with the illuminating field is the modal excitation coefficient (often expressed as modal significance).

To calculate the eigenstates for a target, we need to construct its impedance matrix. The first step is to define the type of problem and select the appropriate surface integral equation for the scattered field. Although there is interest nowadays in platforms made from composite materials, most HF radar studies, including all those calculations included in the present paper, have assumed ship and aircraft targets to behave as closed, perfectly electrically conducting (PEC) targets. Accordingly, the electric field integral equation (EFIE) for the scattered field is rewritten using the PEC boundary condition to yield the relationship between the incident electric field ${\vec{E}}_{inc}$ and the surface current density $\vec{J}$ induced on the target,

$${\vec{E}}_{inc}\left(\vec{r};k,\omega \right)=j\omega \mu \iint_{S}\vec{J}\left({\vec{r}}^{\prime }\right)\frac{e^{-jk\left|\vec{r}-{\vec{r}}^{\prime }\right|}}{4\pi \left|\vec{r}-{\vec{r}}^{\prime }\right|}{dS}^{\prime }+\vec{\nabla }\left(\frac{-1}{j\omega \varepsilon }\iint_{S}\vec{\nabla }\cdot \vec{J}\left({\vec{r}}^{\prime }\right)\frac{e^{-jk\left|\vec{r}-{\vec{r}}^{\prime }\right|}}{4\pi \left|\vec{r}-{\vec{r}}^{\prime }\right|}{dS}^{\prime }\right)\;\equiv \;\tilde{L}\left(\vec{J}\left(\vec{r}\right)\right)$$

where $\vec{r}$ denotes the observation point, ${\vec{r}}^{\prime }$ the source point, and *S* the platform surface.

The impedance operator $\tilde{Z}$, that is, the tangential component of $\tilde{L}$, is obtained in matrix form by discretizing the equation, as first set out in the seminal papers by Harrington and Mautz [67,68]. Unlike Mie scattering, which employs entire-domain basis functions such as spherical harmonics, local, piece-wise functions are used, normally triangular Rao–Willton–Glisson basis functions, which are well suited to modeling complex targets. Inverting the impedance operator $\tilde{Z}$ yields the surface current density $\vec{j}\left(\vec{r}\right)$ on the target by the incident field. The characteristic modes are then obtained by solving the generalized eigenvalue equation

$$\tilde{X}I_{n}=\lambda_{n}\tilde{R}I_{n}$$

where $\tilde{R}$ and $\tilde{X}$ are the real and imaginary parts of $\tilde{Z}$. Importantly, the orthogonality of the characteristic modes is shared by the radiated fields, which then form a basis for the scattering pattern in the far field.

**Figure 38:**
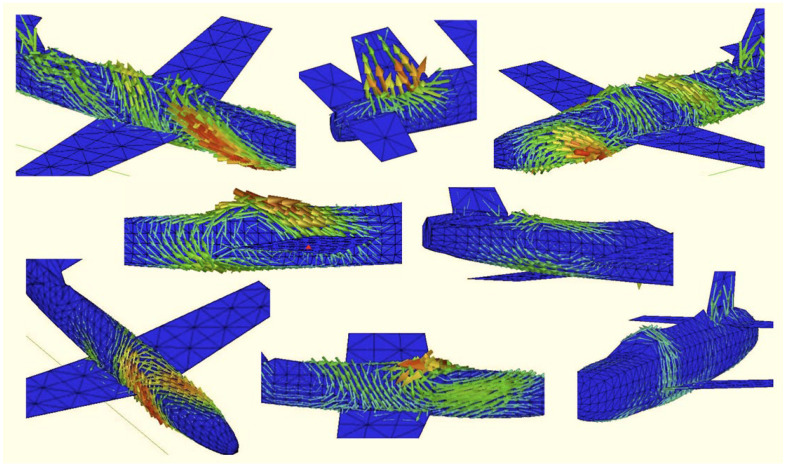
A montage of computed eigen-current distributions—characteristic modes—on the Aermacchi MB 326H aircraft.

Figure 38 is a composite showing a selection of the eigen-currents computed for the Aermacchi MB 26H, revealing that many have localized spatial support.

**Figure 39:**
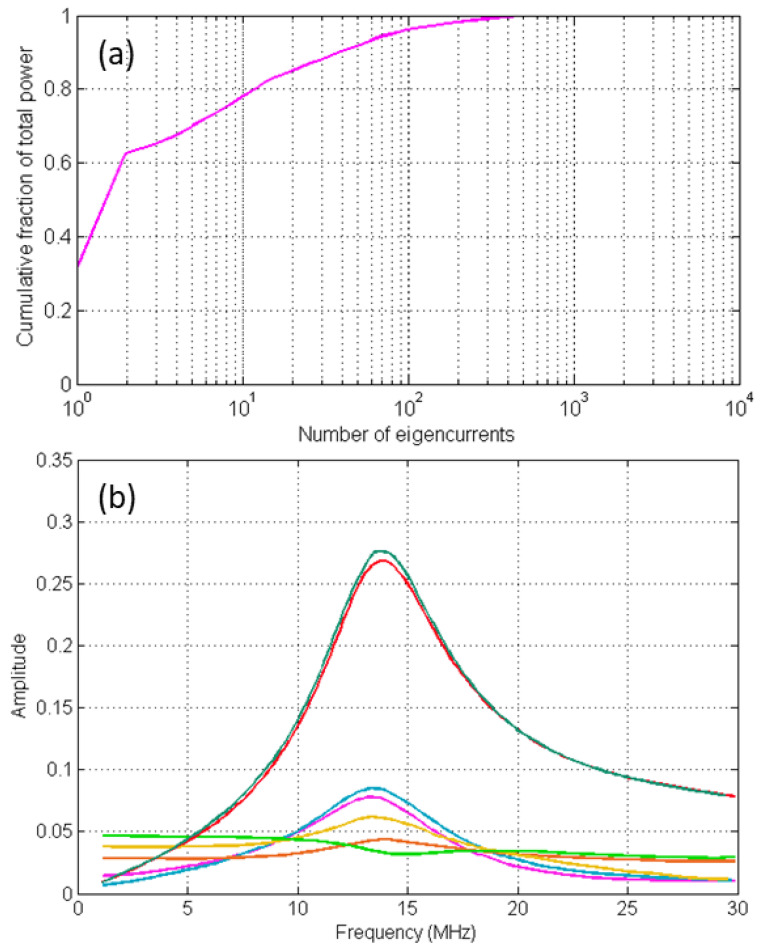
(**a**) Cumulative re-radiated power versus number of models integrated. (**b**) Modal significance of specific modes as radar frequency is varied.

To illustrate the point made above—that in a given scattering scenario, only a few modes contribute significantly—Figure 39a plots cumulative re-radiated power versus number of modes considered in the case treated above, revealing that, in this instance, the first ten modes were responsible for 80% of the re-radiated power, the first two for over 60%. In Figure 39b, the modal significance of the same ten modes is plotted as the radar frequency changes under the same illumination geometry.

The role of characteristic mode analysis in the target classification task is indirect and seemingly applicable only to known target types whose eigenstates are stored in the radar database, i.e., target recognition. Its potential contribution depends on the degrees of freedom accessible to the observing radar, such as frequency agility, polarization, bistatic geometry, and so on. Once the scattering geometry is known, that is, detection has been achieved and coordinate registration has been successful, a small optimal set of probing measurements can be designed that discriminates between likely members of the resident library of known target types. In most respects this is similar to classification based on measurements of accessible elements of the scattering matrix, discussed earlier, but there is a measure of physical insight that may be provided by scenario-specific knowledge. For example, the presence of external stores on aircraft would modify currents flowing on the wings. Then, using radar parameter selection to select dominant modes may enable a degree of confirmation. This is perhaps speculative, and it is unlikely that this kind of information could be exploited by human operators in real time, but, increasingly, AI techniques are being brought to bear such problems. What is more immediately feasible is the exploitation by a platform of knowledge about its own modes to activate real-time adaptive signature control, though we shall not pursue this subject here.

*E.*  Time domain versus frequency domain

The preceding sections have been framed entirely in the frequency domain. There are several practical reasons for this—the narrow bandwidth of accessible HF channels, the long coherent integration times needed to achieve signal gain against noise and clutter, inability to achieve high power densities on the target that could result in significant energy in post-excitation radiating natural modes, the localization in the frequency domain of most target kinematic phase responses, and the nonGaussian external noise background loaded with decaying waveforms from natural phenomena. Techniques such as the singularity expansion method [69] have been contemplated on occasion for use in a hostile electromagnetic environment but rejected after back-of-the-envelope calculations.

Despite this, and in keeping with the principle of keeping an open mind, we recall that Bojarski integrated time-domain response theory with the scattering matrix formulation [70] and made an interesting observation about the possibility of retrieving the matrix through an intervening ionosphere, a subject that was later taken up by others [71] and generalized to the skywave case [72]. Nevertheless, at present, we are not aware of any feasible proposal for implementation in HF over-the-horizon radars as a primary domain for detection or classification. Of course, HF radars rely on signal processing techniques that jump between the two domains [40], or act in the time–frequency domain, to identify propagation distortion mechanisms and compensate for them, but that is a different matter altogether.

### 4.2. Interactive Signatures

Some of the most informative HF signatures are the result of coupling between the target and its environment. We can distinguish two main classes: those where the target’s intrinsic echo is modified by the interaction, and those where the signature of the environment is perturbed by the presence of the target.

A. Kelvin wakes

Within the domain of linearized hydrodynamics in an inviscid fluid, the irrotational flow produced by any pressure distribution within the fluid can be generated by a distribution of Havelock sources, which are point sources possessing the useful attribute that they satisfy the free surface boundary conditions on the mean surface. The velocity potential $\phi \left(x,y,z\right)$ due to a source at coordinate $\left(0,0,-z\right)$ is given by

Gx,y,z=−14πr+14π2R∫−π2π2∫0∞e−iκxcosθ+ysinθκ+κ0sec2θκ−κ0sec2θeκz+ζdkdθ

where $\kappa_{0}=\frac{g}{U^{2}}$.

**Figure 40:**
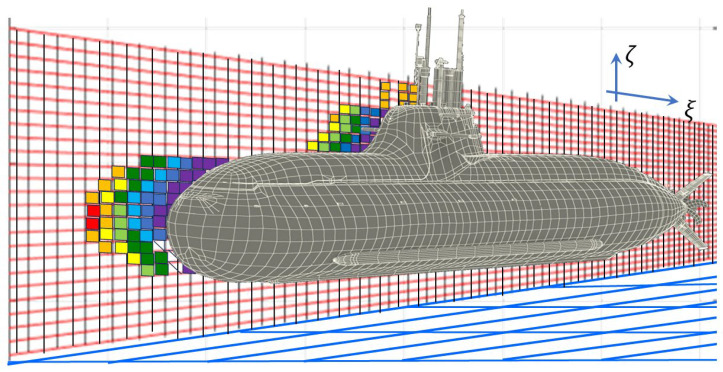
‘Thin ship’ projection of hull sources onto the center plane to simplify the numerical integration. Color is used here to indicate Havelock source strength.

The distinctive Kelvin wake that is generated by a body moving in a fluid at speed U can be modeled by a distribution of these pressure sources over the wetted parts of the body, whether the body is a surface-piercing displacement hull or a fully submerged vessel, i.e., a submarine. The resulting surface displacement at any location $\left(x,y\right)$ is expressible in terms of the integrated response to these contributions once we know the strength of the sources and the shape of the hull that defines the surface of integration. One can address this problem in various approximations. In our previous research, we have followed [66] and used the ‘thin ship’ approximation, which satisfies both our needs [67]. First, the strength of the source at each point on the hull is taken as the gradient of the hull surface along the direction of motion, i.e., the effective area each differential surface element presents to the flow. Second, the surface of integration is approximated by projecting the differential elements of the hull surface onto the vertical center plane $\left(\xi ,\zeta \right)$, hence the ‘thin ship’ terminology, as illustrated in Figure 40 for the case of a submarine.

We then obtain

$$\phi \left(x,y,z\right)=2U\iint_{R}Y_{\xi }\left(\xi ,\zeta \right)G_{x}\left(x-\xi ,y,z;\zeta \right)d\xi d\zeta$$

$$\eta \left(x,y\right)=-\frac{2U^{2}}{\kappa_{0}}\iint_{R}Y_{\xi }\left(\xi ,\zeta \right)G_{x}\left(x-\xi ,y,z;\zeta \right)d\xi d\zeta$$

After some mathematical manipulation (see [73,74] for details), this can be written

ηx,y=R∫−π2π2Aθe−iκ0sec2θxcosθ+ysinθdθ

which we immediately recognize as a spectral representation. This is exactly what we want for our HF radar signature calculation because the scattering theory we use to compute the Doppler spectrum of HF sea clutter uses spectral representations as input. Looking first at the direct problem, in the presence of an ambient directional wave spectrum $S_{0}\left(\vec{\kappa }\right)$, the total wave spectrum becomes $S_{0}\left(\vec{\kappa }\right)+\;S\left(\vec{\kappa }\right)$, where $S\left(\vec{\kappa }\right)={\left|A\left(\theta \left[\vec{\kappa }\right]\right)\right|}^{2}$. Substituting in the standard expression for the HF Doppler spectrum and retaining the terms that do not vanish (except for improbable combinations of vessel and radar parameters [75]),

$$\begin{array}{cc} \tilde{D}\left({\vec{k}}_{scat},{\vec{k}}_{inc};\omega \right)= & \int d{\vec{\kappa }}_{1}F_{1}\left({\vec{k}}_{scat},{\vec{k}}_{inc},{\vec{\kappa }}_{1}\right)S_{0}\left({\vec{\kappa }}_{1}\right)+\\ & +\iint d{\vec{\kappa }}_{1}d{\vec{\kappa }}_{2}F_{2}\left({\vec{k}}_{scat},{\vec{k}}_{inc},{\vec{\kappa }}_{1},{\vec{\kappa }}_{2}\right)S_{0}\left({\vec{\kappa }}_{1}\right)S\left({\vec{\kappa }}_{2}\right) \end{array}$$

To solve the inverse problem for $S\left({\vec{\kappa }}_{2}\right)$, we first need the ambient wave spectrum $S_{0}\left({\vec{\kappa }}_{1}\right)$. This can be retrieved from clutter in neighboring resolution cells assumed free of wakes, as is done routinely in many HF surface wave (and some skywave) radar systems using methods reported in the open literature. Classification is then achieved by solving the linear Fredholm equation and comparing the extracted $A\left(\theta \right)$ with entries in a library.

**Figure 41:**
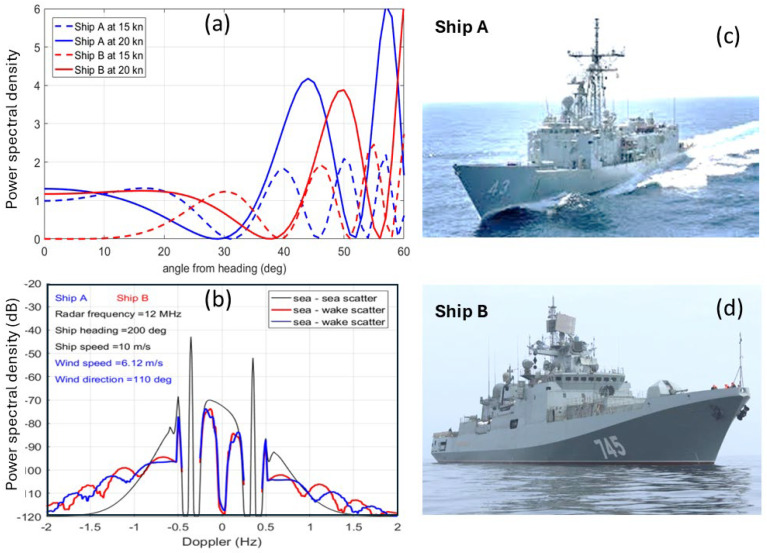
Angular spectra (**a**), and resultant HF Doppler spectra (**b**), computed for two frigates (**c**,**d**).

To demonstrate the sensitivity of wake signatures to hull geometry, consider Figure 41 [76]. In Figure 41a, we show computed wake spectra ${\left|A\left(\theta \right)\right|}^{2}$ of two frigates, computed for two speeds; the vessels are pictured in Figure 41b,d. Figure 41c, compares their respective Doppler spectra at a common speed. The results indicate that classification to type (recognition) is quite achievable in this case.

**Figure 42:**
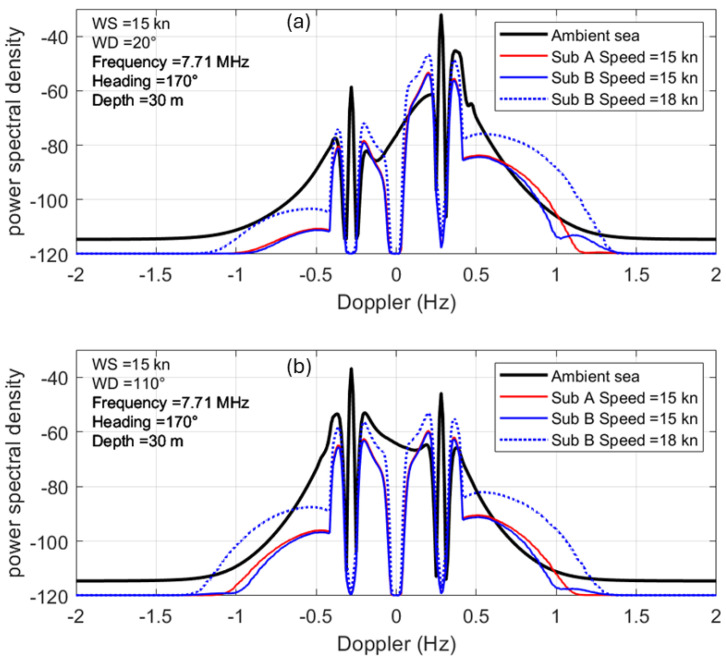
Comparison of Doppler spectra for two submarines at 15 kn and, for one of them, the spectrum at 18 kn. Sea state here is parametrized by wind speed and direction as shown, yielding different background Doppler spectra and hence signature spectra. (**a**) wind direction 20°; (**b**) wind direction 110°. The sensitivity to speed is much greater than that for hull geometry in this case, where the hull lengths differ by about 10%.

As a second example, Figure 42 compares the Doppler spectra of two SSK submarines at the same speed, along with the spectrum of one of them at a speed 20% greater. In this example, the submarines have dimensions differing by only a few percent, so their spectra are effectively indistinguishable, but the 20% change in speed has a strong impact.

Another study looked at the possibility of an HF radar ‘Plimsoll line’, that is, determining vessel loading from its wake [77]. A study of scenario dependence can be found in [78]. We conclude that wakes are a viable classification and recognition domain for surface ships, even when of the same class, but would be more problematic for submarines.

B. Plumes

Rocket exhausts consist of a supersonic stream of combustion products, some ionized, including high concentrations of free electrons. The dimensions and plasma properties of the exhaust plume are functions of the rocket fuel, the nozzle geometry, the velocity of the stream, the ambient pressure, and the speed of the rocket, all parameters of interest for target classification.

Many observations have shown that HF radio waves reflect from the ionized plume, despite the fact that incident radio waves undergo strong attenuation passing through the plume to the rocket body. Several researchers have modeled the rocket–plume composite as a time-invariant but electrically inhomogeneous structure attached to the rocket body. This simplistic model fails to account for the highly Doppler-spread echoes observed in HF radar measurements of rockets in their boost phase, so improved models attribute these components to scattering from plasma inhomogeneities generated by turbulence, as well as continuing combustion of ejected fuel remnants. The large Doppler spread of these echoes can result in energy being aliased across the entire Doppler space, with the extent dependent on the viewing geometry and the radar waveform. Hitherto, interest has focused on the detection problem, so there has been no need to unravel the details of the spectrum.

**Figure 43:**
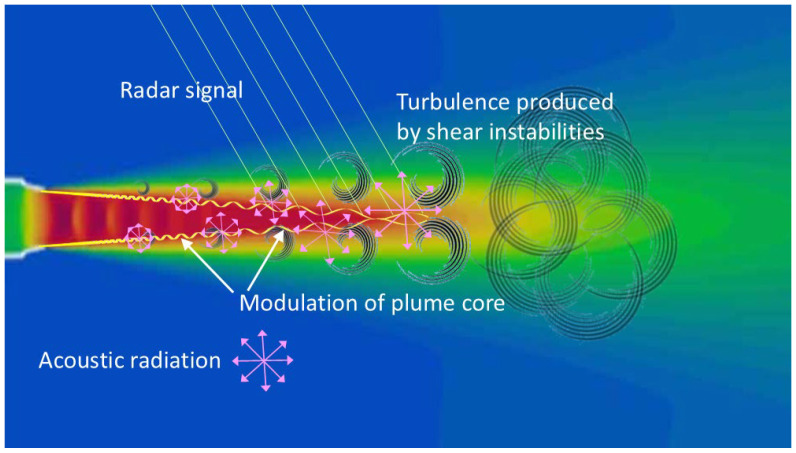
Schematic of the acoustic modulation process.

Recently, it has been conjectured that HF scattering from the plume could reveal enough information about the rocket plume characteristics to recognize the type of motor and hence recognize the vehicle. The basis of this proposition is the recognition that there is an additional mechanism for imposing a Doppler spectrum on the radar echoes. As pictured in the cartoon of Figure 43, the vortices created by shear instabilities along the plume boundary evolve as they are advected along the plume (in the reference frame of the rocket), becoming larger as they travel downstream. The associated pressure fluctuations act as acoustic sources, concentrated in the shear layer.

Measurements of the noise generated during rocket launches, usually with the motivation of assessing effects on people and equipment, have established that the frequency spectrum radiated by these sources moves to lower frequencies as one considers volume elements further downstream, entirely consistent with the source size. Further, the experiments reveal that the strength of the sources rises to a peak near the distance downstream where the shear layer has grown inwards to the core of the plume, where the turbulence levels are amplified due to collision of the unsteady flow features from the circumferential shear layer. In other words, a natural bandpass filter is formed.

In addition to radiating outwards, the highly energetic acoustic radiation also propagates onto the sharp boundary of the highly ionized core. The new hypothesis argues that this would impose a modulation that could manifest itself in radar echoes. The potential for classification then rests on the existence of mathematical or empirical relationships between features in the radar echoes and the plume parameters.

Several such relations exist. First, the laminar core length $L_{c}$ can be related to the nozzle exit diameter $D_{e}$ and the (fully expanded) exit Mach number $M_{e}$ by [79]

$$L_{c}=1.75\;D_{e}{\left(1+0.38M_{e}\right)}^{2}$$

**Figure 44:**
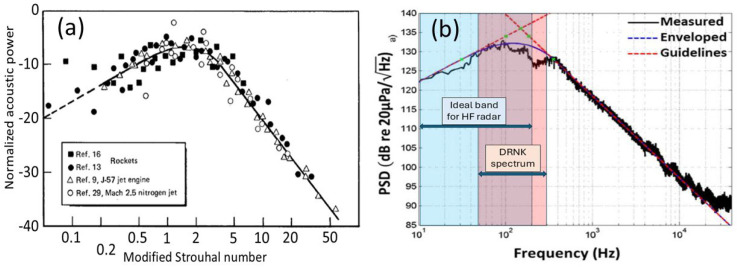
(**a**) Acoustic power vs. (modified) Strouhal number, redrawn from [79]; (**b**) acoustic spectrum of the Orion-50S XLG rocket motor, data from [80] with context overlay.

It is convenient to introduce a dimensionless parameter, the Strouhal number St, which is useful for analyzing oscillating unsteady fluid flow. It expresses the ratio of vibration-to-flow velocities and is defined as

$$St=\frac{f\;L}{U_{e}}$$

where f is the characteristic frequency of vortex shedding on the shear layer boundary, $U_{e}$ is the exit flow velocity, $U_{e}=\;M_{e}c$, with c  the speed of sound, and L is a characteristic length, often set equal to $D_{e}$, but, in our case, we equate it to $L_{c}.$ The utility of the Strouhal number stems from universality of the acoustic spectrum shape vs. St, as shown in Figure 44a; a measurement of the acoustic spectrum of the Orion-50S XLG rocket motor is shown in Figure 44b to illustrate the suitability of HF radar as a sensor in this context.

**Figure 45:**
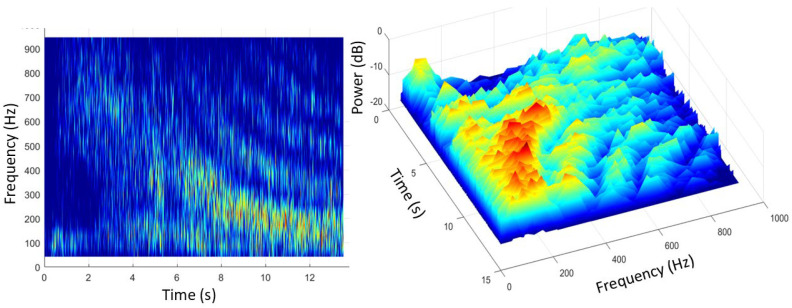
Spectrogram of the DRNK ballistic missile launch, presented in two formats to aid interpretation.

To test this idea, we have carried out spectrum analysis of the audio from an online news video of a ballistic missile launch in the Democratic Republic of North Korea. The spectrogram is presented in Figure 45. It shows a strong fundamental and several harmonics, with a common drift to lower frequencies as the missile ascends. The frequency band has been added to Figure 44b, showing that HF radar is well suited to this mission.

The fundamental frequency in this case is near 480 Hz after 6 s, decreasing to about 180 Hz after 12 s when the rocket is clear of the ground. We have no way of knowing whether the nonlinearity responsible for the harmonics resides in the plume source or the equipment used to record the event. In any case, the frequency spread of several hundred Hz certainly accounts for the aliasing seen in the experimental data to which we have had access.

There are more unknowns than measurements, but as an exercise, we have estimated the core length from the video and this, together with the formulae and the figure, leads to an estimate of the exit velocity and thence to an estimate of the nozzle diameter.

We stress that the proposed mechanism for missile classification or recognition is speculative, and our analysis perhaps overly simplistic. Even so, it is an idea worth pursuing with more experiments, especially given the scenarios in which it might be relevant. Moreover, its inclusion here may provoke thought on the part of the reader, who may conceive other approaches that have eluded the present author.

C. Diffuse scatter

**Figure 46:**
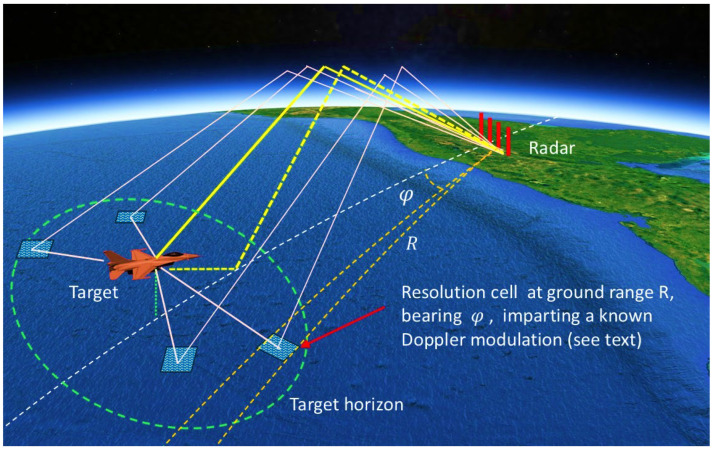
Propagation paths that contribute to the radar observation process. The continuous yellow line represents the 1- ray, the yellow dashed line the 1+ ray, and the grey lines some paths that we term diffuse scattering rays. Such paths are limited to the radar horizon, shown in green. The labeled resolution cell has coordinates known to the radar.

The standard model for skywave detection of airborne targets allows for ground reflection as well as the ionospherically reflected descending rays, so the propagation operators ${\tilde{M}}_{T}^{S}$ and ${\tilde{M}}_{S}^{R}$ need to accommodate four possible two-way signal paths. (The group paths are slightly different, as well as the scattering geometries and Doppler shifts, motivating some researchers to exploit these effects to estimate target altitude, but with very limited success.) An experiment we carried out in the 1980s demonstrated that this basic four-path model is grossly simplistic. Diffuse scatter from the entire region within the elevated target’s horizon can contribute via bistatic surface scatter, as illustrated in Figure 46.

Using the process model, the signal $s_{1}\left(t\right)$ arriving over the one-way path in the absence of diffuse scatter can be written [81]

$$s_{1}\left(t\right)={\tilde{M}}_{{\vec{r}}_{em}}^{\vec{R}}\tilde{\psi }\left({\vec{r}}_{cp}-{\vec{r}}_{em}\right)w\left(t^{\prime }\right)+{\tilde{M}}_{{\vec{r}}_{spec}}^{\vec{R}}{\tilde{m}}_{{\vec{r}}_{em}}^{{\vec{r}}_{spec}}\tilde{\psi }\left({\vec{r}}_{cp}-2{\vec{r}}_{cp}\cdot \hat{z}\hat{z}-{\vec{r}}_{em}\right)w\left(t^{\prime \prime }\right)$$

where $t^{\prime }=t-c^{-1}\int_{{\vec{r}}_{em}}^{\vec{R}}\frac{ds}{\mu }$*,* $t^{\prime \prime }\approx t-c^{-1}\int_{{\vec{r}}_{em}}^{{\vec{r}}_{spec}}\frac{ds}{\mu }-c^{-1}\int_{{\vec{r}}_{spec}}^{\vec{R}}\frac{ds}{\mu }$*,* ${\tilde{m}}_{{\vec{r}}_{1}}^{{\vec{r}}_{2}}$ is the atmospheric propagator, ${\tilde{M}}_{{\vec{r}}_{1}}^{{\vec{r}}_{2}}$ is the skywave propagator, and $\tilde{\psi }\left({\vec{r}}_{2}-{\vec{r}}_{1}\right)$ is the emitter gain in the direction from ${\vec{r}}_{1}$ to ${\vec{r}}_{2}$. The first term is the direct skywave path, the second the ground-bounce path. If the surface is rough, it will support diffuse scatter, so we need an extra term,

$$\iint {\tilde{M}}_{{\vec{r}}_{surf}}^{\vec{R}}\tilde{\sigma }\left(\vec{R}-{\vec{r}}_{surf}{,\vec{r}}_{surf}-{\vec{r}}_{em}\right){\tilde{m}}_{{\vec{r}}_{em}}^{{\vec{r}}_{surf}}\tilde{\psi }\left({\vec{r}}_{surf}-{\vec{r}}_{em}\right)w\left(t^{\prime \prime \prime }\right)d{\vec{r}}_{surf}$$

This diffuse scatter brings a host of benefits, most of which we have reported elsewhere [82]. Here, we focus on the role it can play in target classification. To see this, we linearize the operator expression for the two-way propagation, writing ${\tilde{M}}_{GC}$ to represent the combination of the standard great circle 1- and 1+ rays,

$$\left[{\tilde{M}}_{GC}+{\tilde{M}}_{diff}\right]\tilde{S}\left[{\tilde{M}}_{GC}+{\tilde{M}}_{diff}\right]\approx \left[{\tilde{M}}_{GC}\right]\tilde{S}\left[{\tilde{M}}_{GC}\right]+\left[{\tilde{M}}_{GC}\right]\tilde{S}\left[{\tilde{M}}_{diff}\right]+\left[{\tilde{M}}_{diff}\right]\tilde{S}\left[{\tilde{M}}_{GC}\right]$$

Both $\left[{\tilde{M}}_{GC}\right]\tilde{S}\left[{\tilde{M}}_{GC}\right]$ and $\left[{\tilde{M}}_{GC}\right]\tilde{S}\left[{\tilde{M}}_{diff}\right]$ terms arrive at the receiver along the great circle through radar and target, so, by spatial filtering, we can isolate the last term. In a more explicit form, the received signal to be used for classification appears as a distribution over group range, azimuth, and Doppler, where the Doppler shift includes the contribution from the target velocity $\vec{V}\cdot \left({\vec{r}}_{cell}-{\vec{r}}_{targ}\right)$ and the modulation from the sea clutter Doppler spectrum (only the first-order terms are significant in our application). The amplitude factor follows immediately from the directional wave spectrum, retrievable from the radar data as mentioned earlier. The remaining unknown is the aircraft altitude h. This can be estimated from the limits of the azimuthal spread of the echo, $\left[\varphi_{max}^{left},\;\varphi_{max}^{right}\right]$, even when the distribution is asymmetric.

**Figure 47:**
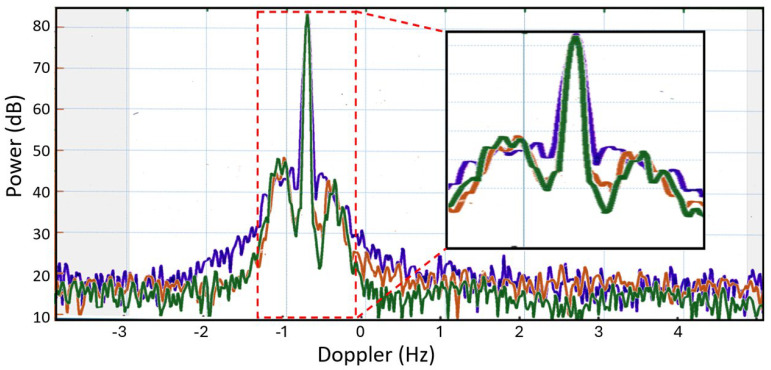
Signals received via one-way propagation over the sea from a boat transmitting a linear FM waveform. Brown curve: range = 90 km, speed = 0; green curve: range = 100 km, speed = 0; blue curve: range = 100 km, speed = 12 knots. Aligned in main peaks.

Diffuse scatter in HF surface wave radar presents a similar but less complicated scattering configuration, mentioned in Section 3.2. A detailed theoretical model was reported in [44] and validated in various experiments for the static emitter case. This work was extended by experiments in 1999 with the Iluka HFSWR that measured the one-way propagation operator by radiating from a vertical monopole antenna on a small boat in the target zone at ranges extending to 120 km from the receiving array [82]. The contributions from diffuse scatter processes were observed with the boat stationary, moving towards, and moving away from the receiver and in tight circles for calibration of the radiation pattern. Measurements confirmed the role of intermediate scattering from the sea surface, as illustrated in Figure 47. Here, the curves shown in green and brown were recorded from the stationary boat at two ranges, 90 km and 100 km, while that in blue was recorded with the boat traveling towards the radar at 12 knots. For ease of comparison the boat Doppler shift has been removed.

The slight broadening of the direct signal in the blue curve, relative to the others, is indicative of the greater hull motions when underway. The high dynamic range shown does not provide the fine resolution in power needed to reveal any associated small change in peak amplitude, but that is of secondary concern here—we are presenting experimental evidence for the presence and importance of diffuse scatter contributions on HFSWR paths.

Again, the curves show the power received on the shore from the transmitter on the boat, not an echo from a transmitter on the shore. The latter case, involving two-way propagation, can be modeled from the one-way propagation measurements by convolution over the spatial and temporal domains.

The classification potential of the diffusely scattered echoes follows from the bistatic scattering cross section of the vessel, which can be retrieved from the range–Doppler map.

D. Dynamic signatures

A conjugate to the generation of wakes by a moving ship is the ship’s response to forcing applied to the wetted surface of the ship by the ambient ocean waves. Figure 10, presented in Section 4.1, identifies the six degrees of freedom that describe translational and rotational rigid body motions. Ships are also subject to bending and torsional stresses that are capable, in rough seas, of modifying the ship geometry enough to have an effect on the HF radar signature. Here, we shall consider only the rigid body motions.

To compute the radar signatures associated with these motions, we need models for three separate dynamical processes. First, there is the ambient sea state, customarily represented by a directional wave spectrum of deep-water gravity waves, with secondary effects arising from nonlinear interactions. Parametric models have been developed to represent such spectra, though these usually need to be supplemented with models for swell. Second, there is the response of the ship to the forcing from these waves. This is not straightforward because the spectrum felt by the ship is that observed in its own frame of reference, not the spectrum measurable in geocentric coordinates.

When a platform traveling at speed $\vec{U}$ encounters a wave with wavevector $\vec{\kappa }$ and intrinsic angular frequency $\omega_{in}=\sqrt{g\left|\vec{\kappa }\right|}$, the angular frequency observed by the ship—the encounter frequency—is given by

$$\omega_{en}=\omega_{in}-\vec{\kappa }\cdot \vec{U}=\omega_{in}-\frac{\omega_{in}^{2}}{g}\left|\vec{U}\right|cos\theta$$

The wave energy spectrum $S\left(\omega_{en}\right)\;$ that is experienced by the ship is then related to the geocentric energy spectrum $S\left(\omega_{in}\right)\;$ by the Jacobian,

$$S\left(\omega_{en}\right)=\frac{g}{g-2\omega_{in}\left|\vec{U}\right|cos\theta }\cdot \;S\left(\omega_{in}\right)$$

For some degrees of freedom, notably pitch and roll, the wave slope spectrum is the primary actor. In this case,

$$S_{sl}\left(\omega_{en}\right)=\frac{g}{g-2\omega_{in}\left|\vec{U}\right|cos\theta }\cdot \;S_{sl}\left(\omega_{in}\right)=\frac{g}{g-2\omega_{in}\left|\vec{U}\right|cos\theta }\cdot \;\frac{\omega_{in}^{4}}{g^{2}}\;S\left(\omega_{in}\right)$$

Within the domain of validity of a linearized approach, the response of a ship to a spectrum of waves is the sum of the responses to the component sinusoidal waves. For each ship degree of freedom, there exists a measure of its response to a given encountered wave, a transfer function defined in the frequency domain. Usually interest focuses on the magnitude, not the phase of the response, so the squared magnitude of that transfer function is used to characterize the sensitivity of that degree of freedom; this is known as the Response Amplitude Operator (RAO) for that degree of freedom [83]. It follows that the variance of the associated motion of the ship is given by the product of the RAO and the encounter frequency spectrum,

$$S_{\iota }\left(\omega_{en}\right)={RAO}_{i}\cdot S\left(\omega_{en}\right)\mathrm{or}\;S_{\iota }\left(\omega_{en}\right)={RAO}_{i}\cdot S_{sl}\left(\omega_{en}\right)$$

**Figure 48:**
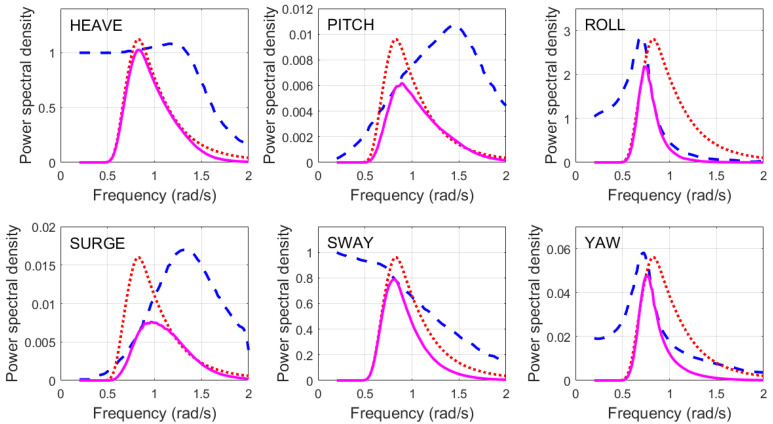
Nominal responses for the six degrees of freedom of the FFG 7, computed for a beam sea, at 10 knots sailing speed, using rescaled RAO model estimates from [84] and a JONSWAP wave spectrum evaluated at a wind speed of 20 knots. The blue dashed lines show the RAOs, the red dotted lines the sea wave spectrum, and the magenta full lines the products of the RAOs and the sea wave spectrum. PSD units are m^2^/(rad/s).

**Figure 49:**
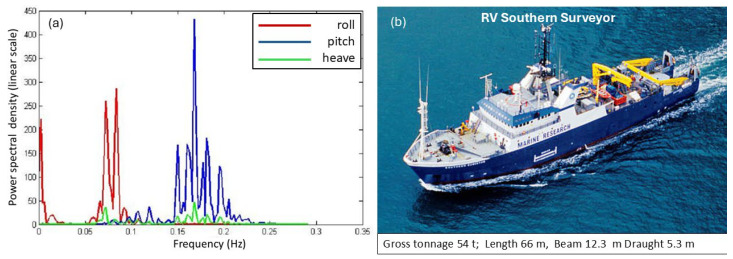
(**a**) Measured spectra of roll, pitch, and heave from (**b**) RV Southern Surveyor, recorded in the Southern Ocean under sea state 3 conditions, 2003, showing how the RAOs filter the ambient spectrum [85].

We illustrate this idea in Figure 48. The importance of this to target characterization with HF radar stems from the fact that the RAOs can be precalculated and stored for known ships, while the radar itself can measure $S\left(\vec{\kappa }\right)$ and hence $S\left(\omega_{in}\right)$, which is easily converted to $S\left(\omega_{en}\right)$ or $S_{sl}\left(\omega_{en}\right)$ once the ship heading has been estimated. However, what the radar measures is not $S_{\iota }\left(\omega_{en}\right)$ but, as the process model dictates, a proportional quantity that includes as a factor the ship RCS (or scattering matrix), which varies as the ship changes its orientation relative to the radar. Further, if a certain depth of modulation of RCS is observed and associated with ship roll, say, based on the oceanographic measurements, it is hard to discriminate between (i) a large roll angle of a ship with a small RAO and (ii) a small roll angle for a ship with a large RAO. It would seem plausible to suggest that this ambiguity might be resolved by high-fidelity electromagnetic modeling of ship scattering characteristics as a function of aspect. We continue to explore this avenue, guided by measurements such as Figure 49a, which shows a superposition of onboard measurements of $S_{roll}\left(\omega_{en}\right)$, $S_{pitch}\left(\omega_{en}\right),$ and $S_{heave}\left(\omega_{en}\right)$ recorded on a CSIRO oceanographic vessel, pictured in Figure 49b. As roll can exceed 25° for frigates in high sea states, and pitch may reach 10° or more in head seas, the resulting aspect modulation of the radar scattering signature is likely to be observable in such conditions.

**Figure 50:**
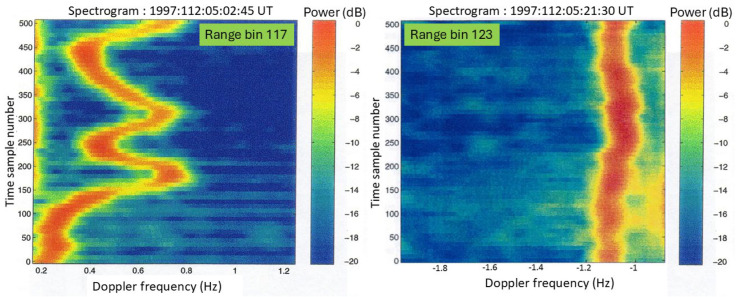
Spectrograms of (**a**) a maneuvering motor launch and (**b**) the same boat maintaining a constant speed and direction in a low sea state.

To illustrate the feasibility of the basic idea of what might be termed a form of micro-Doppler, consider Figure 50, which shows two spectrograms, each recorded over 128 s at a frequency of 3.90 MHz [86]. On the left, the trace shows the echo from a small motor launch that is maneuvering in a roughly sinusoidal pattern about an inbound course. We can see a correlated variation in amplitude as its aspect changes. On the right, we see the trace from the same boat as it attempts to motor at constant speed in a linear course in sea state 1. We can see fluctuations in Doppler despite the best efforts of the pilot to maintain constancy, surely deserving of the term micro-Doppler.

**Figure 51:**
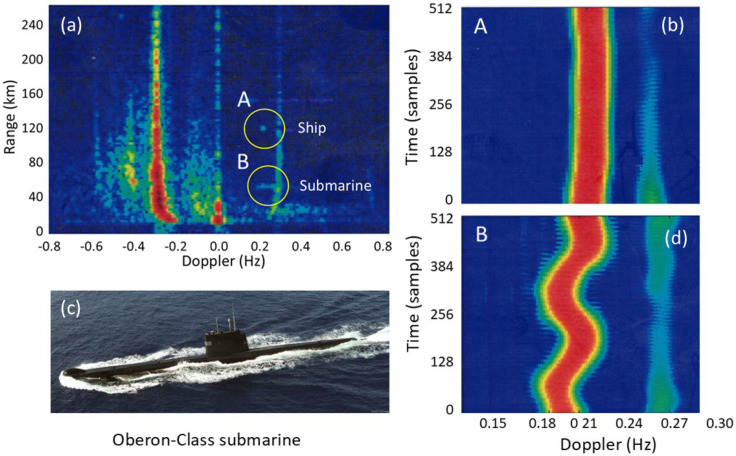
HF echo from a submarine steaming on the surface in a following swell. (**a**) ship and submarine echoes identified on range–Doppler map; (**b**) Oberon-Class submarine; (**c**) time–frequency analysis of ship echo; (**d**) time–frequency analysis of submarine echo showing modulation.

A different situation is shown in Figure 51, which shows the trace from an Oberon-Class submarine steaming on the surface at an intended constant speed but exhibiting phase modulation that we would normally attribute to platform-controlled Doppler. However, on this occasion, the vessel was experiencing significant pitch and surge motions due to a strong following swell from the Southern Ocean, so the phase modulation could contain the effects of both advective Doppler variation over the phase of the swell and RCS modulation as a function of pitch angle, manifesting as a phase modulation. For short vessels, the latter is observable, but it is unlikely in this case, judging by the results shown in Figure 33d,e.

This historical data is not accessible for refined analysis of echo power variations but, given a little environmental information, we could obtain a crude estimate of the consistency of the surge hypothesis by modeling the speed-over-ground (SOG) measured by the radar as the sum of the speed-through-water (STW) and the bulk fluid motion associated with the swell of period $T_{S}$. For deep-water waves, the echo modulation period $T_{E}$ (for both surge and RCS) is then given by

$$T_{E}=\frac{g}{2\pi }\frac{T_{S}^{2}}{\left(\frac{gT_{S}}{2\pi }-STW\right)}$$

The results in Figure 51d show an oscillation with a period of ~16 s, which is in reasonable agreement with the prediction from the formula given typical submarine speeds on the surface and the prevailing environmental conditions—heavy swell with a period in the range 10–13 s.

### 4.3. Behavioral Signatures

While the intrinsic radar signatures of aircraft and ships, discussed in the preceding sections, are the primary sources used for target characterization, observable parameters related to vehicle motions, environmental conditions, geographical context, and inference about likely mission objectives can offer valuable clues.

A. Kinematics

Ships and aircraft are free to adapt their speed and course according to need, but there are fundamental constraints imposed by design and the operating environment. For aircraft, the trade-off between parasitic drag, which increases with speed, and induced drag, which decreases with speed, results in a U-shaped efficiency curve. The minimum speed increases with altitude, but, to a good approximation, the curve retains its essentially quadratic form. If the aircraft may be presumed to be operating with fuel efficiency in mind, its type and altitude will be coupled; knowledge of one will inform the other. Commercial aircraft must take into account many factors, but, even so, their speeds at normal cruising altitude are remarkably consistent, ranging from 0.82 M to 0.86 M (M = Mach number) according to type. We have no information on military aircraft, but the laws of physics still apply. What can be said is that all HF radar operators learn to recognize many aircraft from their cruising speeds.

Another consideration is change of direction. To turn, an aircraft rolls or banks then increases the lift; so over the course of a turn, it presents to the radar an aspect in the form of a curve in azimuth–elevation space, which maps into the polarization matrix functional dependence on those parameters. In the case of a skywave radar, the incident polarization may be unknown but will be constant for the time it takes for the maneuver, so recognition will depend on aspect sensitivity, which increases with radar frequency.

For ships, the situation is somewhat different—speed is significantly influenced by sea state once it passes a certain hull-dependent threshold. Up to that point, optimum efficiency is still coupled to speed via the Froude number variation of wave resistance and frictional drag. If a vessel’s speed departs from one that is efficient, that, in itself, is informative, indicating that some other consideration is active. As for maneuvers, ship design is not the only factor—deck-mounted cargo, large quantities of liquids, ship stability in seas, and presence of other traffic all constrain vessel dynamics. The risk of instability is substantially increased in stern quarter seas, so vessels choosing courses that avoid this geometry may be inadvertently signaling their metacentric stability limits, which provide information on beam, hull shape, and mass distribution.

B. Intentification

We coined this term in 1999 to reflect the fact that aircraft and ships travel with a purpose, and this objective may reasonably be assumed to fall within the capabilities of the platform, its equipment, and its endurance, as well as the prevailing situation in the theater under observation. If a vessel departs from a Great Circle path between common standard shipping lane waypoints or changes course and speed near critical facilities, some associated intent may be responsible. Examples of this have appeared in recent times with sabotage to submarine communication cables in the Baltic Sea and elsewhere. HF radar can easily detect the changes in ship velocity associated with such activities, as we have verified using AIS data from ships suspected of those acts.

Another clue to platform type may sometimes be found in correlated movements. For example, if two vessels are being tracked while spatially well separated, any synchronous changes of course by both vessels suggest cooperation, from which further inferences might be made.

These arguments may seem to signal the need for classification rather than a means of achieving it, but any information that narrows the search space, or even modifies the a priori likelihood of a particular type of platform, has an impact on target characterization procedures and outcomes.

C. Spatial clues

If aircraft altitude estimation is within the capabilities of the observing radar, that may eliminate some candidate platform types, especially when combined with kinematic information. Theory and experiment have shown that one of the untapped abilities of HF skywave radar is the estimation of aircraft position, course, speed, altitude, and class from diffuse surface scatter [81]. In a similar vein, the geographical setting, bathymetry, locations of support facilities, presence of extreme weather, exploitable currents, and mesoscale eddies all contribute to the maritime scenario unfolding in the region being surveyed by the radar.

### 4.4. Responsive Signatures

HF radars providing support to cooperating platforms can take advantage of IFF measures carried by those assets to identify them and hence eliminate some of the detected targets from the need for assigning radar resources to the classification task. In this regard, it is important to point out that IFF can be achieved without special hardware by pre-arranged kinematic or passive modulation techniques.

## 5. Implementation and Accessibility

With many radars, the timeline is almost invariably filled with primary missions of detection and tracking, so the reallocation of resources to implement target characterization procedures will involve trade-offs. These may take the form of changes to radar frequency, waveform, coherent integration time, and scan pattern within the existing task schedule or demand the insertion of entirely new tasks directed at measuring environmental conditions such as the ionospheric channel polarization bandwidth, the availability of adequately spaced dual-frequency propagation channels, post-compensation phase path stability, and the ocean directional wave spectrum. As described in the preceding sections, this kind of information plays an essential role in the generation of some signatures and hence their retrieval and interpretation.

**Figure 52:**
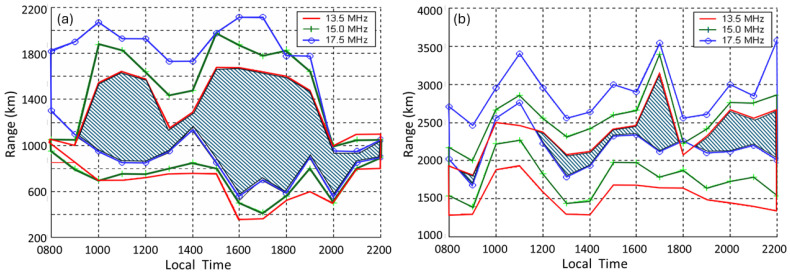
Joint availability of multiple frequencies over the course of 14 h. (**a**) E-mode propagation; (**b**) F-mode propagation. Shaded areas indicate availability of all frequencies shown.

It bears repeating that some signatures are accessible only under a restricted range of target-dependent environmental conditions. To illustrate, first we consider the availability of propagation channels able to support multi-frequency skywave measurements. Figure 52a plots the ground scatter footprint in range of three carrier frequencies, recorded over a 14 h period on 28 January 2001 using the Jindalee radar at a single bearing. The chosen frequencies—13.5, 15, and 17.5 MHz—span a band sufficiently wide to offer a reasonable prospect of target discrimination. The shaded region shows the range–time window of simultaneous availability of all three frequencies when reflection from the E-region is employed. Figure 52b shows the corresponding information when F-mode propagation is selected. It is evident that, in operational practice, one would need to monitor conditions and seize opportunities as they arise.

**Figure 53:**
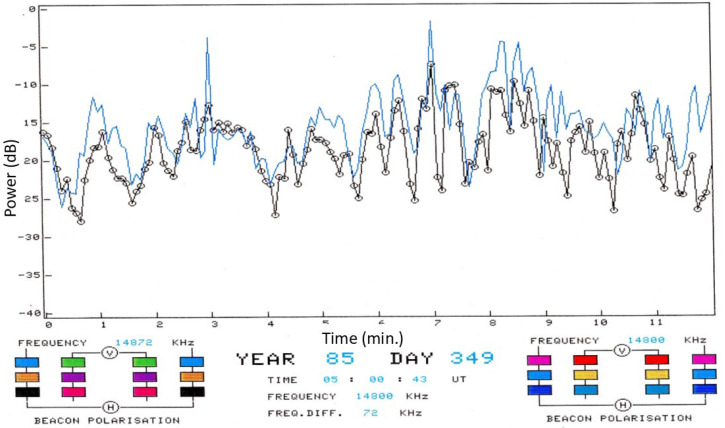
Polarization fading sequences recorded at two spaced frequencies, 72 kHz apart. Black line with markers represents 14872 kHz, blue line 14800 kHz.

Second, we present in Figure 53 a record of echo strength from a discrete scatterer, namely a transponder at a range of 1260 km. In this experiment, the ability of the Jindalee radar to function as two independent half-radars was exploited to transmit and receive signals on two carrier frequencies simultaneously, at a selectable spacing, with the bandwidth of each signal set to 10 kHz.

**Figure 54:**
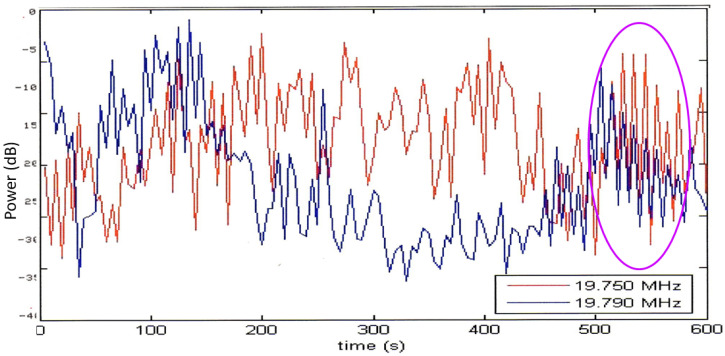
Polarization fading sequences recorded at two spaced frequencies, 40 kHz apart.

In this example, the frequencies were 14.800 and 14.872 MHz, so the spacing was 72 kHz. The figure, which plots only the E − E two-way propagation mode, though many modes and different transponders were active, shows a high correlation between the fading patterns, which arise from time-varying polarization in the ionosphere. One can conclude that, on this occasion, a waveform with a swept bandwidth of 72 kHz would not experience a significantly varying polarization state at the target, so a meaningful compromise between range resolution and polarization constancy is achievable. In contrast, Figure 54 shows a measurement over 600 s, during which the two transmitted frequencies, separated by only 40 kHz, display quite different fading patterns, rendering even moderately wide bandwidth waveforms unprofitable for some purposes. Of incidental interest in this example is the appearance of a common polarization modulation with a period of ~10.1 s, associated with a Pc-3 ULF micropulsation, indicated by the magenta ellipse.

The message is clear: whatever the potential of some multi-frequency or polarimetric observations for target characterization via skywave radar, their successful implementation is reliant on the availability of suitable propagation conditions and the ability to find them in real time and adapt the radar parameters appropriately.

Beyond these readily accessible measures, radars may be augmented with ancillary facilities such as additional transmitting or receiving antennas, some degree of polarimetric capability, and versatile calibration resources including buoys and nonlinear devices. For skywave radars, the familiar propagation management support facilities such as sounders may need to adopt more general forms. For example, HF radars need advice on channels in which to transmit; most skywave radars are scrupulous in avoiding other users of the spectrum, but there is always a level of background noise against which detections must be made. Present-day spectrum monitors often provide a measure of the level of this noise but not its rejectability, i.e., the level to which it might be further reduced by signal processing.

In addition to hardware extensions, most advanced techniques require access to mission-oriented databases. These include those that describe the relevant construction and performance attributes of the known targets of interest, along with the outputs from special processing of the generalized sounder and spectrum monitoring data.

The prospective roles of artificial intelligence in target characterization are at present so nebulous that nothing tangible can be added to early thoughts in that direction [86], but, judging by the speed at which AI is enhancing task performance in scores of disciplines, any radar implementation today should provide data pathways that can be used by AI tomorrow.

## 6. Conclusions

The surveillance capabilities of HF radar have been amply demonstrated over the past half century, during which performance has been enhanced by several orders of magnitude, but the ‘added value’ of target characterization has failed to keep pace. There are many reasons for this, not least the challenges posed by propagation and the fact that target dimensions fall in the resonance scattering regime at HF, but one might also point to the lack of a suitably comprehensive framework within which the target characterization task can be formulated and used to guide operational practice.

In this paper, we have presented a taxonomy of signature domains that spans all the prospective means of HF radar discrimination that have been reported in the literature, along with some that have hitherto escaped attention. Within each domain, we have described the relevant physical mechanisms and illustrated them with experimental results, where available for publication, and with computer model outputs in other cases.

The implementation of some of these methods requires radar parameter values and degrees of freedom that are not universally shared by present-day HF radars. Moreover, some approaches may be viable only under particularly favorable environmental conditions. Nevertheless, every one of the ideas proposed here is feasible across an operationally meaningful range of scenarios.

Target characterization at HF is a highly ambitious goal, as implied by the modest progress that has been reported to date. The need to distinguish between platforms that are visibly very similar, without the advantage of high spatial resolution, obliges us to expand the task into as many domains as are accessible in any given circumstance, so that discrimination can proceed in a space with maximum dimensionality. The results presented in this paper confirm that there are many possibilities.

We have not attempted to categorize methods into groups applicable to individual radar configurations—skywave, surface wave, line-of-sight, hybrid, and so on. To do so might foreclose on imaginative concepts that extend the ideas presented here.

Raising unrealistic expectations is not in the long-term interest of proponents of this technology. Yet, as we have endeavored to demonstrate, physics suggests that much remains to be exploited by the HF radar community. A measure of boldness is surely appropriate to stimulate the development and operational implementation of all the techniques that have the potential to contribute.

## Data Availability

The original contributions presented in this study are included in the article. Further inquiries can be directed to the corresponding author.
